# Reduced structural connectivity in cortico-striatal-thalamic network in neonates with congenital heart disease

**DOI:** 10.1016/j.nicl.2020.102423

**Published:** 2020-09-15

**Authors:** Megan Ní Bhroin, Samy Abo Seada, Alexandra F. Bonthrone, Christopher J. Kelly, Daan Christiaens, Andreas Schuh, Maximilian Pietsch, Jana Hutter, J-Donald Tournier, Lucillio Cordero-Grande, Daniel Rueckert, Joseph V. Hajnal, Kuberan Pushparajah, John Simpson, A. David Edwards, Mary A. Rutherford, Serena J. Counsell, Dafnis Batalle

**Affiliations:** a Centre for the Developing Brain, School of Biomedical Engineering & Imaging Sciences, King’s College London, London, UK; b Trinity College Institute of Neuroscience and Cognitive Systems Group, Discipline of Psychiatry, School of Medicine, Trinity College Dublin, Ireland; c Department of Electrical Engineering (ESAT/PSI), KU Leuven, Leuven, Belgium; d Department of Computing, Imperial College London, London, UK; e Biomedical Image Technologies, ETSI Telecomunicación, Universidad Politécnica de Madrid & CIBER-BBN, Madrid, Spain; f Paediatric Cardiology Department, Evelina London Children's Healthcare, London, UK; g Congenital Heart Disease, Evelina London Children's Hospital, London, UK; h Department of Forensic and Neurodevelopmental Science, Institute of Psychiatry, Psychology & Neuroscience, King’s College London, London, UK

**Keywords:** Congenital heart disease, Infant, Brain, Diffusion MRI, Graph Theory, dHCP

## Abstract

Impaired brain development has been observed in newborns with congenital heart disease (CHD). We performed graph theoretical analyses and network-based statistics (NBS) to assess global brain network topology and identify subnetworks of altered connectivity in infants with CHD prior to cardiac surgery. Fifty-eight infants with critical/serious CHD prior to surgery and 116 matched healthy controls as part of the developing Human Connectome Project (dHCP) underwent MRI on a 3T system and high angular resolution diffusion MRI (HARDI) was obtained. Multi-tissue constrained spherical deconvolution, anatomically constrained probabilistic tractography (ACT) and spherical-deconvolution informed filtering of tractograms (SIFT2) was used to construct weighted structural networks. Network topology was assessed and NBS was used to identify structural connectivity differences between CHD and control groups. Structural networks were partitioned into core and peripheral nodes, and edges classed as core, peripheral, or feeder. NBS identified one subnetwork with reduced structural connectivity in CHD infants involving basal ganglia, amygdala, hippocampus, cerebellum, vermis, and temporal and parieto-occipital lobe, primarily affecting core nodes and edges. However, we did not find significantly different global network characteristics in CHD neonates. This locally affected sub-network with reduced connectivity could explain, at least in part, the neurodevelopmental impairments associated with CHD.

## Highlights

• A subnetwork with reduced structural connectivity was identified in infants with CHD.
• This subnetwork comprised regions predominantly in the cortico-striatal-thalamic network.
• Core nodes and core edges were mostly affected in this subnetwork.
• Global network features were not significantly different in CHD group.

## 1 Introduction

Congenital heart disease is the most common congenital disorder, with an estimated incidence of 6–8 per 1000 live births (van der Bom et al., 2011). Recent advances in surgical procedures and perioperative care have led to a significant decrease in mortality rates and most children born with CHD now survive to adulthood (Wren and O’Sullivan, 2001). As mortality rates have declined, research efforts have shifted to understanding and improving neurodevelopmental outcomes and quality of life for survivors of CHD. Neurodevelopmental impairments are common (Marino et al., 2012) and can involve several developmental domains, including cognition, executive function, motor and language skills, and behavioural impairments (Gaynor et al., 2015, Latal, 2016).

Magnetic resonance imaging (MRI) studies have identified a high incidence of acquired brain lesions and impaired brain development in infants with CHD (Glauser et al., 1990, Miller et al., 2004, Miller et al., 2007, Hinton et al., 2008, Ortinau et al., 2012, von Rhein et al., 2015, Kelly et al., 2017, Kelly et al., 2019a, Kelly et al., 2019b, McQuillen et al., 2006, Claessens et al., 2016). Brain dysmaturation in this population encompasses reduced total and regional brain volume (Ortinau et al., 2012, von Rhein et al., 2015); impaired cortical gyrification (Kelly et al., 2017, Claessens et al., 2016) and microstructural development (Kelly et al., 2019a); reduced *N*-acetylaspartate (NAA) to choline ratios and elevated mean diffusivity in deep grey and white matter; and reduced white matter fractional anisotropy (Miller et al., 2007).

Recent advances in MRI have made it possible to describe global organization properties of structural and functional brain networks through the application of graph theoretical approaches (Bullmore and Sporns, 2009). Graph theory analysis applied to connectivity matrices can extract important network features (Hagmann et al., 2012) and has been used to investigate alterations in brain development in preterm infants (van den Heuvel et al., 2015, Batalle et al., 2017). The presence of a high capacity central core, or rich club, has been observed in adults (van den Heuvel and Sporns, 2011) and in infants (Ball et al., 2014). Previous reports in populations at-risk of neurodevelopmental impairment have found that core-connectivity is maintained and peripheral (i.e. local) connectivity is disrupted (Fischi-Gomez et al., 2016, Karolis et al., 2016, Batalle et al., 2017, Ball et al., 2014). In neonates with CHD, recent studies have demonstrated reduced functional connectivity (De Asis-Cruz et al., 2018) and alterations in structural network topology pre-operatively (Schmithorst et al., 2018). However, it is not clear whether altered structural network topology in infants with CHD prior to surgery can be explained by disruptions to core-peripheral connections.

In this study we used high angular resolution multi-shell diffusion imaging to characterise brain organisation of the structural network in newborns with CHD and controls. Our aims were to (i) determine global and local network features in newborns with CHD and healthy controls, (ii) assess core and peripheral network organisation in both groups and (iii) identify subnetworks of altered connectivity in infants with CHD using network-based statistics (NBS) (Zalesky et al., 2010).

## 2 Methods

The project was approved by the National Research Ethics Service West London committee

(CHD 07/H0707/105; Controls 14/LO/1169). Informed written consent was obtained from the parents of all participants before scanning.

### 2.1 Participants

The study included fifty-eight infants with critical or serious CHD. Critical CHD was defined as hypoplastic left heart syndrome, pulmonary atresia with intact ventricular septum, transposition of the great arteries, interruption of the aortic arch and all infants dying or requiring surgery within the first 28 days of life with the following conditions: coarctation of the aorta; aortic valve stenosis; pulmonary valve stenosis; tetralogy of Fallot; pulmonary atresia with ventricular septal defect; total anomalous pulmonary venous connection. Serious CHD was defined as any cardiac lesion not defined as critical, which requires cardiac catheterisation or surgery, or results in death before age one (Ewer et al., 2011, Kelly et al., 2019b). Exclusion criteria included suspected or confirmed chromosomal abnormality or congenital syndrome, neonatal surgery before recruitment (excluding cardiac catherization procedures), suspected congenital infection, or arterial ischaemic infarction on MRI. Twelve infants with transposition of the great arteries underwent balloon atrial septostomy prior to MRI. Thirty-one infants with CHD were on a prostaglandin infusion to maintain ductal patency at the time of scan and none required mechanical ventilation at the time of scanning.

A control group of 116 healthy infants was matched to the CHD group by gestational age (GA) at birth, post-menstrual age (PMA) at scan and sex. Healthy infants were recruited contemporaneously from the postnatal ward at St Thomas’ Hospital as part of the developing Human Connectome Project (dHCP) (http://www.developingconnectome.org/). Table 1 shows the demographic characteristics of the two groups.

**Table 1:** Demographic Characteristics of the CHD and Control Cohorts.

Variable	Newborns with CHD (n = 58)	Control newborns (n = 116)	*p*-value
Gestational age at birth, weeks	38.43 (38–38.86)	38.71 (37.86–39.29)	0.1579
Postmenstrual age at scan, weeks	39.07 (38.57–39.71)	39.14 (38.43–39.71)	0.884
Male sex, n (%)	33 (57%)	66 (57%)	1
Birth weight (kg)	3.06 (2.77–3.45)	3.11 (2.71–3.46)	0.6162
Birth head circumference (cm)	33.65 (32.4–35)	34 (33–35)	0.5306
Primary Heart lesion - n (%)			
Transposition of the great arteries	27 (46%)	−	
Coarctation of the aorta	12 (21%)	−	
Tetralogy of Fallot	7 (12%)	−	
Pulmonary stenosis	4 (7%)	−	
Hypoplastic left heart syndrome	3 (5%)	−	
Pulmonary atresia	3 (5%)	−	
Truncus arteriosus	1 (2%)	−	
Tricuspid atresia	1 (2%)	−	

Values presented as median (interquartile range) unless otherwise stated. *p*-values calculated using Mann-Whitney *U* test.

### 2.2 Data acquisition

MR imaging was performed on a Philips 3 Tesla system (Best, The Netherlands) located in the neonatal intensive care unit in the Evelina Newborn Imaging Centre at St. Thomas Hospital using a 32-channel neonatal head coil and neonatal positioning device (Hughes et al., 2017). Pulse oximetry, temperature, electrocardiography and respiratory rate were monitored during the MR examinations which were supervised by a paediatrician trained in MR procedures. Infants were scanned in natural sleep and provided with ear protection comprising earplugs moulded from silicone based putty placed in the external auditory meatus (President Putty, Coltene Whaledent, Mahwah, NJ), neonatal earmuffs (MiniMuffs, Natus Medical Inc, San Carlos,CA) and an acoustic hood placed over the infant. T1-weighted images were acquired using the following parameters; repetition time (TR) = 11 ms, echo time (TE) = 4.6 ms, flip angle = 9°, voxel size = 0.8 × 0.8 × 0.8 mm. T2-weighted images were acquired using a multislice turbo echo sequence: TR = 12 s, TE = 156 ms, flip angle = 90°, in-plane resolution = 0.8 mm, slice thickness = 1.6, overlap = 0.8 mm. Susceptibility-weighted imaging (SWI) was acquired using a spoiled gradient-recalled echo sequence: TR = 3.2 s, TE = 25 ms, flip angle = 12°, voxel size = 0.45x0.45x1.8 mm. Diffusion MRI was acquired with a high angular resolution diffusion (HARDI) multi-shell protocol designed specifically for the neonatal brain; TR = 3.8 s, TE = 90 ms, volumes = 300, multiband factor = 4, sensitivity encoding E: 1.2; resolution: 1.5 × 1.5 × 3 mm with 1.5 mm slice overlap, diffusion gradient encoding: b = 0 s/mm^2^ (n = 20), b = 400 s/mm^2^ (n = 64), b = 1000 s/mm^2^ (n = 88), b = 2600 s/mm^2^ (n = 128) with interleaved phase encoding (Hutter et al., 2018).

### 2.3 Qualitative MRI analysis

MR images were reported by two neonatal neuroradiologists. All images were subsequently rereviewed to ensure consistency, and lesions classified as focal arterial ischaemic stroke (AIS), white matter injury (WMI), cerebellar haemorrhage or intraventricular haemorrhage as described previously (Kelly et al., 2019b). The location and properties of lesions on T1 and T2-weighted imaging, SWI and apparent diffusion coefficient (ADC) map were recorded. WMI was classified into normal (no WMI), mild (≤3 foci and all ≤ 2 mm), moderate (>3 and ≤ 10 foci or any > 2 mm) or severe (>10 foci) (Beca et al., 2013). Overall each baby was categorised into one of four brain injury groups; normal, mild (intraventricular haemorrhage, and/or cerebellar haemorrhage ≤ 2 mm, and/or mild WMI), moderate (cerebellar haemorrhage > 2 mm and/or moderate WMI) and severe (severe WMI) (Kelly et al., 2019b).

### 2.4 Pre-processing and network construction

All T2-weighted images were motion corrected and reconstructed to a 0.8 mm isotropic resolution (Cordero-Grande et al., 2018), bias field corrected (Tustison et al., 2010), brain extracted (Smith, 2002) and segmented into white matter (WM), grey matter (GM), deep grey matter (DGM), cerebrospinal fluid (CSF) and cerebellum using an extension of the Draw-EM algorithm (Makropoulos et al., 2014, Makropoulos et al., 2018). Parcellation was performed using the anatomical automatic labelling (AAL) atlas (Tzourio-Mazoyer et al., 2002) mapped to neonates (Shi et al., 2011) resulting in 93 cortical, subcortical and cerebellar regions, and manually corrected using a high-resolution dHCP atlas (Schuh et al., 2018). The parcellation was normalised from template space to native T2-weighted space using the diffeomorphic symmetric image normalization method (SyN) in the Advanced Normalization Tools (ANTs) software package (Avants et al., 2008). Tissue maps and parcellation were registered using rigid registration with the Image Registration Toolkit (IRTK) (Studholme et al., 1999) from each infant’s T2-weighted native space to diffusion native space with average b = 0 volumes used as the target.

Diffusion MRI was reconstructed to an isotropic resolution of 1.5 mm, denoised (Cordero-Grande et al., 2019, Veraart et al., 2016), Gibbs ringing artefacts suppressed (Kellner et al., 2016), and corrected for motion and image distortion using spherical harmonics and radial decomposition (SHARD) (Christiaens et al., 2019). Using a group averaged response function sampled in WM and in CSF from control infants, tissue and free water orientation distribution functions (ODFs) were estimated using multi-shell multi-tissue constrained spherical deconvolution (Jeurissen et al., 2014) and subsequently normalized to obtain quantitative measures of density (Raffelt et al., 2017). The normalised tissue ODFs were used to generate 10 M streamlines from probabilistic tracking using anatomically constrained probabilistic tractography (ACT) (Smith et al., 2012) with biologically accurate weights (SIFT2) (Smith et al., 2015, Tournier et al., 2019).

The fibre density SIFT2 proportionality coefficient (µ) for each subject was obtained to achieve inter-subject connection density normalisation, and structural connectivity (SC) was considered as the weighted sum (SIFT2*µ) of streamlines connecting each pair of regions, resulting in the construction of a 93x93 structural connectivity matrix for each subject.

### 2.5 Network measures

Graph theoretical analyses were carried out in order to assess topological properties of individual weighted networks using functions from the Brain Connectivity Toolbox (BCT) (Rubinov and Sporns, 2010) for Matlab (version R2018b). Global functioning of brain networks was characterized by assessing infrastructure, integration and segregation. Network infrastructure was assessed by network density which measures the proportion of observed edges relative to the number of possible edges (Kaiser, 2011) and average strength which describes the connectivity of a node to other nodes by summing all of the edge weights in the network. In order to assess brain network integration, we measured global efficiency which takes the inverse of the average shortest path length between nodes to provide a measure of relative parallel information exchange between distributed regions across the network (Achard and Bullmore, 2007, Latora and Marchiori, 2001, Latora and Marchiori, 2003). Local efficiency, which represents network segregation and measures the efficiency of information exchange among neighbouring nodes was also calculated. Local efficiency reflects the fault tolerance of the network, by assessing how well each subnetwork exchanges information following the removal of random nodes from the network (Achard and Bullmore, 2007). Each of these network features were calculated in original reconstructed networks (“raw” networks). In addition, we summarised how the organisation of structural connectivity changes with increasing age at scan by assessing the relationship of infrastructure, integration and segregation with PMA at MRI scan. Calculation of all network characteristic formulations were based on definitions by Rubinov and Sporns (2010).

### 2.6 Core and periphery partitioning and local characteristics

We partitioned structural connectivity networks into two distinct groups of nodes, which consisted of a core and periphery structure. This was achieved using an adapted version of the Kernighan-Lin algorithm for graph partitioning (Borgatti and Everett, 2000, Newman, 2006) available in the BCT toolbox (Rubinov and Sporns, 2010). This produces an optimal core-periphery structure such that core nodes are well-connected to other core and periphery nodes, while periphery nodes are not well connected to each other, and results in a set of highly connected and strongly interconnected hubs and a sparsely connected brain periphery. We defined a common core/periphery structure as nodes belonging to the core/periphery partitioning in 90% of subjects. To quantify the goodness of fit of the core/periphery partition we calculated the coreness statistic which estimates the degree of separation between core and peripheral nodes (Borgatti and Everett, 2000). For regional nodal characteristics of the core and periphery structure, we considered nodal efficiency measuring how well a specific node is integrated within the network via its shortest paths (Latora and Marchiori, 2001, Achard and Bullmore, 2007), and nodal strength which represents the sum of edge weights connected to each node.

### 2.7 Network based statistics

The structural connectomes were then evaluated with the network-based statistics (NBS) toolbox for MATLAB, which detects differences in structural connectivity between groups using permutation testing (Zalesky et al., 2010). NBS considers multiple comparisons when identifying subnetworks that exhibit significant structural differences between groups. We used a general linear model (GLM) with 10,000 permutations and multiple comparison correction (*p* = 0.05) when comparing the extent (i.e. total number of connections) between groups. In NBS, correction for multiple comparisons is carried out by cluster-based thresholding whereby connected components of a network are treated as a cluster. We used the primary test-statistics threshold (*t* = 3.1) to define a set of supra-threshold connections in which the connections with a test statistic value exceeding this threshold are considered significant. Since NBS results are highly dependent on the primary test-statistics threshold, we tested a range of values (*t* = 2.5–3.5) and show the results for *t* = 3.1. The NBS analysis was controlled for relevant covariates including sex, GA at birth, PMA at scan, and overall brain injury score.

### 2.8 Statistical analysis

Group comparisons were examined with a general linear model (GLM) using the multivariate analysis of covariance (MANCOVAN) toolbox by William Gruner (https://www.mathworks.com/matlabcentral/fileexchange/27014-mancovan) in Matlab R2018b (Mathworks Inc., Mattick, USA) with sex, GA at birth, PMA at scan, and overall brain injury score as fixed effects. Partial Spearman’s correlations were used to assess the association between graph theory features and PMA at scan, while also controlling for sex, GA at birth and overall brain injury score. All analyses were carried out using Matlab R2018b. BrainNet Viewer was used for visualizations of nodes and edges (Xia et al., 2013).

## 3 Results

### 3.1 Clinical characteristics

The analysis included 174 newborn infants, which comprised of 58 neonates with CHD scanned prior to surgery and 116 age-matched healthy controls. There was a higher proportion of infants with CHD with mild (*p* = 0.0161) and severe WMI (*p* = 0.0443). Four (7%) infants with CHD had cerebellar haemorrhage. There were no cases of cerebellar haemorrhage or severe WMI in control infants. Details of imaging findings in both groups are shown in Table 2.

**Table 2:** Characteristics and MRI findings of the infants.

Variable	Newborns with CHD (n = 58)	Control newborns (n = 116)	*p*-value
Cerebellar haemorrhage, n (%)	4 (7%)	0	**0.0042**
White matter Injury (WMI), n (%)			
Normal	40 (69%)	101 (87%)	**0.0042**
Mild	11 (19%)	8 (7%)	**0.0161**
Moderate	5 (9%)	7 (6%)	0.4028
Severe	2 (3%)	0	**0.0443**
Overall brain injury score, n (%)			
0 – Normal	37 (64%)	101 (87%)	**0.0004**
1 – Mild	13 (22%)	8 (7%)	**0.0031**
2 – Moderate	6 (10%)	7 (6%)	0.3080
3 – Severe	2 (3%)	0	**0.0443**

*p*-values calculated using Chi-squared statistics.

### 3.2 Global network features

**Fig. 1:**
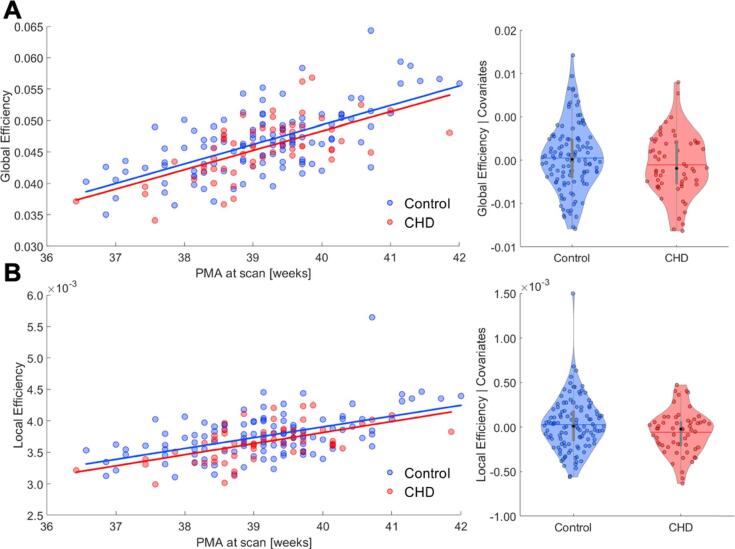
Global graph theory characteristics in CHD and control group. Relationship between global (*A*) and local efficiency (*B*) with post-menstrual age (PMA) at scan. Regression line indicates significant positive relationship. Violin plots representing distribution of residuals after correcting for PMA at scan, GA at birth, sex and overall brain injury score. Global efficiency did not differ between groups, whereas local efficiency in controls was significantly higher compared to CHD neonates (*p* = 0.033). However, after removing the outlier, statistical significance was lost.

Increasing postmenstrual age at MRI scan was positively associated with average network strength (ρ = 0.3429, *p* < 0.001), global efficiency (ρ = 0.3935, *p* < 0.001) (Fig. 1A), and local efficiency (ρ = 0.3533, *p* < 0.001) (Fig. 1B), but negatively associated with total network density (ρ = -0.3955, *p* < 0.001). No difference between groups was found when assessing infrastructure for total network density (*p* = 0.242) and average network strength (*p* = 0.177). Furthermore, when investigating network integration, we found no difference in global efficiency *(p* = 0.150) (Fig. 1A) between the two cohorts. Analysis of network segregation revealed that local efficiency was significantly higher in controls compared to neonates with CHD [MANCOVA: *F*_(5, 168)_ = 4.60, *p =* 0.033] (Fig. 1B). However, after removing one outlier from the analysis of local efficiency (defined as 3 scaled median absolute deviations (MAD) away from the median), statistical significance was lost (*p* = 0.103).

### 3.3 Core/Periphery partitioning and local characteristics

**Fig. 2:**
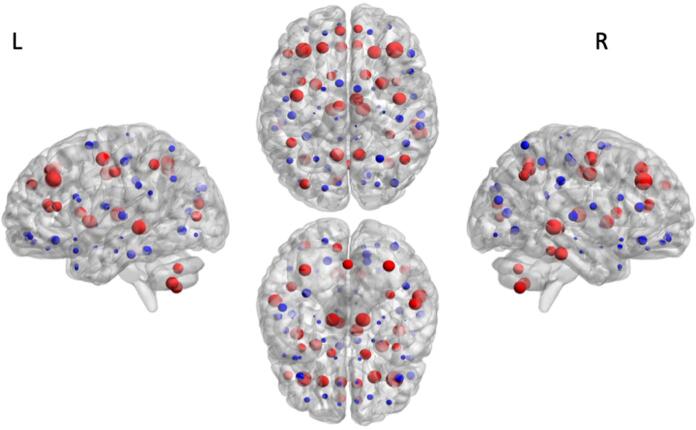
Distribution of core/periphery nodes common in CHD and control networks. In a network with a core-periphery organization core nodes (red) are well-connected to each other and nodes in the periphery (blue) are not well connected to one another. From left to right, lateral view of the left hemisphere, transverse view of both hemispheres (superior, inferior) and lateral view of the right hemisphere. Size of nodes represented by nodal strength. Images were generated using the BrainNet Viewer software (Xia et al. 2013). (For interpretation of the references to colour in this figure legend, the reader is referred to the web version of this article.)

A common core/periphery structure was defined for the whole study population (see Table 3 for a full list of core and periphery nodes, represented in Fig. 2) as described in Section 2.6. This analysis revealed a core comprised of 34 regions, which included the insula, precuneus, superior frontal cortex as well as subcortical thalamus and putamen. The coreness statistic was calculated for each subject and found to be significantly higher in CHD compared to control neonates [MANCOVA: *F*_(5, 168)_ = 5.01, *p =* 0.026].

**Table 3:** List of core (n = 34) and peripheral nodes (n = 59) common in CHD and control networks.

**Core nodes**		**Peripheral nodes**	
Precentral gyrus left & right	Middle temporal gyrus left & right	Orbitofrontal cortex (superior) left & right	Superior occipital gyrus left & right

Superior frontal gyrus (dorsal) left & right	Inferior temporal gyrus right	Orbitofrontal cortex (middle) left & right	Middle occipital gyrus right
Middle frontal gyrus left & right	Cerebellum left & right	Inferior frontal gyrus (opercular) left & right	Fusiform gyrus left
Inferior frontal gyrus (triangular) left	Vermis	Inferior frontal gyrus (triangular) right	Inferior occipital gyrus left & right
Superior frontal gyrus (medial) left & right		Orbitofrontal cortex (inferior) left & right	Postcentral gyrus left & right
Insula left & right		Rolandic operculum left & right	Superior parietal gyrus left & right
Anterior cingulate gyrus left & right		Supplementary motor area left & right	Inferior parietal lobule right
Median cingulate and paracingulate gyrus left & right		Olfactory left & right	Supramarginal gyrus left & right
Calcarine cortex left		Orbitofrontal cortex (medial) left & right	Angular gyrus left
Middle occipital gyrus left		Rectus gyrus left & right	Paracentral lobule left & right
Fusiform gyrus right		Posterior cingulate gyrus left & right	Pallidum left & right
Inferior parietal lobule left		Hippocampus left & right	Heschl gyrus left & right
Angular gyrus right		Parahippocampal gyrus left & right	Superior temporal gyrus left & right
Precuneus left & right		Amygdala left & right	Temporal pole (superior) left & right
Caudate left & right		Calcarine cortex right	Temporal pole (middle) left & right
Putamen left & right		Cuneus left & right	Inferior temporal gyrus left
Thalamus left & right		Lingual gyrus left & right

**Fig. 3:**
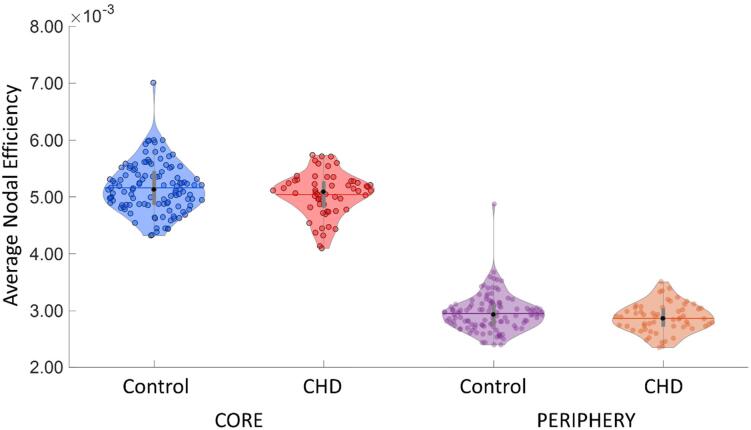
Average nodal efficiency of core and periphery nodes in CHD and control groups. Average nodal efficiency in CHD is significantly lower in core (*p* = 0.046) and periphery (*p* = 0.035). However, after removing outliers, no significant difference is found.

We carried out a regional analysis in order to investigate whether core and/or peripheral (i.e. local) connections were affected in the CHD group. This was carried out by assessing group differences in nodal characteristics of core and peripheral structures and allowed us to determine whether nodes from either structures were affected as a consequence of CHD. Nodal efficiency of the core structure was significantly lower in CHD neonates compared to controls [MANCOVA: *F*_(5, 168)_ = 4.03, *p =* 0.046] (Fig. 3). Similarly, in the peripheral structure nodal efficiency was significantly lower in CHD neonates compared to controls [MANCOVA*: F*_(5, 168)_ = 4.51, *p* = 0.035] (Fig. 3). However, for both core and periphery average nodal efficiency, after removing the aforementioned outlier, statistical significance was lost. We found no difference in nodal strength in core and peripheral structures between groups.

### 3.4 Subnetwork of brain regions with reduced connectivity in CHD

**Fig. 4:**
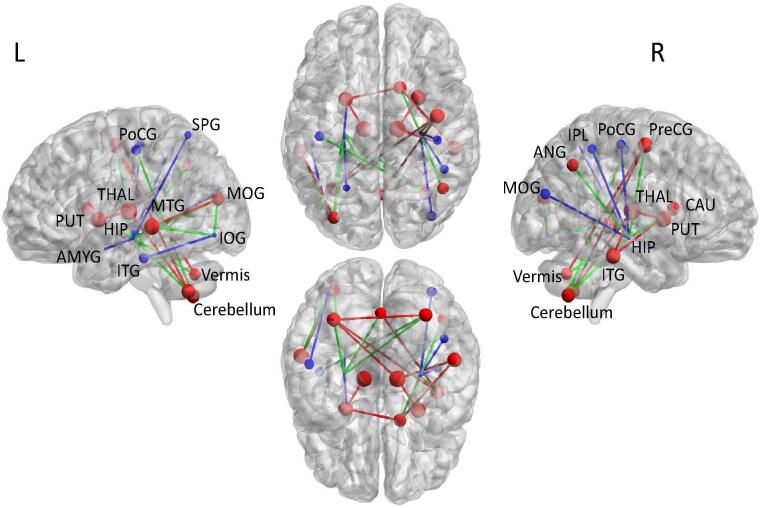
Illustration of NBS-derived subnetwork with reduced structural connectivity in CHD neonates compared to controls. From left to right, lateral view of the left hemisphere, transverse view of both hemispheres (superior, inferior) and lateral view of the right hemisphere. Each dot and line represents a node and edge in which structural connectivity is reduced in CHD neonates. Red and blue regions correspond to nodes from the core and peripheral structure, respectively. Size of nodes represented by nodal strength. Edge colours correspond to core-core edges in red (core), peripheral-peripheral edges in blue (peripheral) and core-peripheral edges in green (feeder). Abbreviations: PreCG, precentral gyrus; HIP, hippocampus; AMYG, amygdala; MOG, middle occipital gyrus; IOG, inferior occipital gyrus; PoCG, postcentral gyrus; SPG, superior parietal gyrus; IPL, inferior parietal lobule; ANG, angular gyrus; CAU, caudate; PUT, putamen; THAL, thalamus; MTG, middle temporal gyrus; ITG, inferior temporal gyrus. Images were generated using the BrainNet Viewer software (Xia et al. 2013). (For interpretation of the references to colour in this figure legend, the reader is referred to the web version of this article.)

We further assessed whether specific sub-networks were affected in the CHD group using NBS. We found a single subnetwork comprising 23 nodes sharing 26 edges with reduced connectivity in CHD (Fig. 4). This distributed network included connections between the vermis, bilateral hippocampus, thalamus, cerebellum, putamen, posterior cingulate gyrus, middle occipital gyrus, and left amygdala, inferior occipital gyrus, superior parietal gyrus, middle temporal gyrus, and right precentral gyrus, inferior parietal lobule and caudate. This subnetwork comprised of 11 nodes situated in the right, and 11 nodes within the left hemisphere, as well as the vermis. Affected edges of the subnetwork included 9 right intra-hemispheric, 9 left intra-hemispheric, 6 inter-hemispheric connections, and 2 connecting to the vermis. We then assessed whether regions identified in the subnetwork belonged to either the core or periphery structure and assessed which connection type was most affected. Edges were defined as; core connections between core nodes, peripheral connections between peripheral nodes; and feeder connections between peripheral and core nodes. From the 23 nodes with reduced structural connectivity in infants with CHD, 13 were core nodes (out of a total of 34 core nodes, 38.23%), while 10 were peripheral nodes (out of a total of 59 peripheral nodes, 16.94%). Of the 26 edges with reduced connectivity in CHD, 10 (38.46%) were core, 10 (38.46%) were feeders and 6 (23.07%) were peripheral. We did not identify any subnetworks with increased connectivity in the CHD group compared with controls. Table 4 lists the nodes and edges comprising the disconnected sub-network in neonates with CHD and is represented in Fig. 4.

**Table 4:** Subnetwork with reduced structural connectivity in CHD neonates.

**#**	**Node**	**Node network**	**#**	**Edge**	**Edge type**	***t*-value**
1	Precentral gyrus right	Core	1	Hippocampus left − Middle occipital gyrus left	Feeder	3.11
2	Middle occipital gyrus left	Core	2	Precentral gyrus right − Hippocampus right	Feeder	3.12
3	Angular gyrus right	Core	3	Caudate right − Inferior temporal gyrus right	Core	3.13
4	Caudate right	Core	4	Precentral gyrus right − Cerebellum left	Core	3.13
5	Putamen left	Core	5	Middle occipital gyrus left − Middle temporal gyrus left	Core	3.15
6	Putamen right	Core	6	Middle occipital gyrus left − Inferior occipital gyrus left	Feeder	3.17
7	Thalamus left	Core	7	Hippocampus left − Superior parietal gyrus left	Peripheral	3.17
8	Thalamus right	Core	8	Inferior occipital gyrus left − Middle temporal gyrus left	Feeder	3.29
9	Middle temporal gyrus left	Core	9	Hippocampus right − Inferior parietal lobule right	Peripheral	3.31
10	Inferior temporal gyrus right	Core	10	Hippocampus left − Amygdala left	Peripheral	3.34
11	Cerebellum left	Core	11	Hippocampus left − Cerebellum left	Feeder	3.36
12	Cerebellum right	Core	12	Hippocampus right − Middle occipital gyrus right	Peripheral	3.4
13	Vermis	Core	13	Postcentral gyrus left − Cerebellum right	Feeder	3.43
14	Hippocampus left	Peripheral	14	Thalamus right − Cerebellum left	Core	3.51
15	Hippocampus right	Peripheral	15	Putamen right − Thalamus right	Core	3.52
16	Amygdala left	Peripheral	16	Thalamus right − Inferior temporal gyrus right	Core	3.52
17	Middle occipital gyrus right	Peripheral	17	Hippocampus right − Angular gyrus right	Feeder	3.58
18	Inferior occipital gyrus left	Peripheral	18	Precentral gyrus right − Vermis	Core	3.70
19	Postcentral gyrus left	Peripheral	19	Inferior occipital gyrus left − Inferior temporal gyrus left	Peripheral	3.72
20	Postcentral gyrus right	Peripheral	20	Hippocampus right − Postcentral gyrus right	Peripheral	3.77
21	Superior parietal gyrus left	Peripheral	21	Putamen left − Thalamus left	Core	3.77
22	Inferior parietal lobule right	Peripheral	22	Caudate right − Putamen left	Core	3.81
23	Inferior temporal gyrus left	Peripheral	23	Cerebellum left − Cerebellum right	Core	3.81
			24	Hippocampus left − Vermis	Feeder	3.93
			25	Hippocampus left − Cerebellum right	Feeder	3.97
			26	Hippocampus right − Caudate right	Feeder	4

## 4 Discussion

This study provides evidence for altered structural connectivity in a cortico-striatal-thalamic sub-network in newborns with CHD prior to surgery.

Human brain development is characterised by rapid changes in brain structure due to myelination, synaptogenesis and dendritic arborisation (Kostović and Jovanov-Milošević, 2006). Studies assessing structural brain organisation have revealed that the brain tends to be more segregated in the fetal (Song et al., 2017) and preterm brain (Batalle et al., 2017, Zhao et al., 2019, van den Heuvel et al., 2015, Ball et al., 2014, Tymofiyeva et al., 2012, Brown et al., 2014), improving in integration capacity during the first years of life (Hagmann et al., 2010, Dennis and Thompson, 2013, Huang et al., 2015, Tymofiyeva et al., 2013, Khundrakpam et al., 2013, Fan et al., 2011, Yap et al., 2011) due to the development of long-range association fibres which support higher cognitive functions (Huang and Vasung, 2014, Bullmore and Sporns, 2012).

In infants with CHD pre-operatively, a recent diffusion tensor imaging (DTI)-based connectivity study identified reorganisation of global network topology of structural brain networks (Schmithorst et al., 2018), reporting disruptions to cost and global efficiency (integration). However, in our population we found that newborns with CHD had preserved network infrastructure and integration, while disruption in segregation had a small effect size, and was not statistically significant after the removal of outliers. To further understand the discrepancy with previously reported differences, we carried out a regional analysis to assess whether there were subtle alterations to core-peripheral connectivity. Core or rich club regions are brain hubs that form the backbone of the brain network (van den Heuvel et al., 2012, van den Heuvel and Sporns, 2013) allowing integration of specialized cortical regions (Senden et al., 2014). Our analysis revealed core regions that included the insula, precuneus, superior frontal cortex as well as subcortical thalamus and putamen, consistent with previous descriptions of rich club regions described in adults (van den Heuvel and Sporns, 2011) and neonates (Ball et al., 2014). However, similar to our global network results, we found only a small effect associated with CHD, which did not reach statistical significance after removing outliers.

Notwithstanding, we did find altered connectivity at the sub-network level using NBS, revealing one sub-network of structural connections in which connectivity strengths were significantly reduced in neonates with CHD compared to controls. This distributed network comprised connections predominantly in the cortico-striatal-thalamic network, involving regions in the basal ganglia, amygdala, hippocampus, cerebellum, vermis, and cortical regions in the temporal and parieto-occipital lobe. Our structural findings correspond with a recent resting state fMRI study of pre-operative CHD newborns which identified one subnetwork with reduced functional connectivity involving the putamen, caudate and thalamus (De Asis-Cruz et al., 2018). The thalamus is an important site for the integration of networks supporting the ability to modulate behaviour (Haber and Calzavara, 2009), while the striatum, the main input station of the basal ganglia, is associated with the regulation of motor (Lehericy et al., 2005) and cognitive functioning (van Rooij et al., 2015). Disruptions to cortico-thalamic circuits have been previously reported in children with ADHD (Castellanos, 1997, Casey et al., 2007), and dysfunction of the cortico-striatal-thalamic network has been described in Parkinson’s disease patients (Hacker et al., 2012) and cognitive disorders including, bipolar disorder (Chen et al., 2006) and Tourette syndrome (Makki et al., 2009) and in school age children born extremely prematurely with intrauterine growth restriction (IUGR) (Fischi-Gómez et al., 2015, Eixarch et al., 2016). Moreover, recent reports have documented subcortical morphological abnormalities in CHD patients across their lifespan (Wong et al., 2017, von Rhein et al., 2014, von Rhein et al., 2015, Owen et al., 2014, Ortinau et al., 2012). DTI and magnetic resonance spectroscopy (MRS) evaluation of term born newborns with CHD preoperatively revealed reduced fractional anisotropy (FA) in subcortical white matter tracts, and increased average diffusivity (AD), decreased ratio of NAA to choline and increased lactate to choline ratio in the basal ganglia and thalamus (Miller et al., 2007). Additionally, reduced volumes of subcortical structures have been reported in fetuses with hypoplastic left heart syndrome (HLHS) (Clouchoux et al. 2013) and in newborns (Owen et al., 2014), and are associated with impaired cognitive abilities in adolescents with CHD (von Rhein et al., 2014).

We also observed reduced structural connectivity in a number of regions that are important for memory, cognition, executive function and attention. Specifically, we observed reduced structural connectivity in the hippocampus and amygdala in infants with CHD, structures that are important in memory (Scoville and Milner, 1957, Tulving, 2002), cognition (Squire, 2004), and emotion regulation (LeDoux, 1996), respectively. We also found reduced structural connectivity in the precentral gyrus. Reduced FA in the precentral white matter has previously been shown to correlate with deficits in executive function and inattention/hyperactivity symptoms in adolescents with CHD (Rollins et al., 2014). We identified reduced structural connectivity in the cerebellum and vermis, regions that are crucial for motor control, coordination (Morton and Bastian, 2004) and evidence suggests that the cerebellum may also play an important role in cognitive processing and emotional control (Schmahmann and Caplan, 2006).

Of note, core regions were affected more than peripheral in the subnetwork with reduced structural connectivity in infants with CHD. Additionally, we showed decreased feeder connections with core regions of the right precentral gyrus, angular gyrus, and caudate, left middle occipital and temporal gyrus and bilateral cerebellum. Core components play a key role in the efficient integration of information processing among distant brain regions, therefore disruptions to core connectivity have a widespread effect on information transfer in the brain (van den Heuvel and Sporns, 2011, van den Heuvel et al., 2012). Although it is widely believed that damage to core connections severely impacts the global efficiency to the network (van den Heuvel and Sporns, 2011), we did not find such alterations in global efficiency between CHD and matched controls. While studies of preterm infants suggest core connections are relatively preserved (Fischi-Gomez et al., 2016, Karolis et al., 2016, Batalle et al., 2017), our findings are consistent with a previous functional connectivity study in infants with CHD which found that rich club regions were primarily affected in a subnetwork of nodes with reduced functional connectivity (De Asis-Cruz et al., 2018). Our findings suggest core connections in regions associated with important aspects of behaviour including cognition, behaviour modulation, motor control, and emotion regulation, are more vulnerable in newborns with CHD.

Our study has some limitations. Our CHD cohort is heterogeneous. Infants had a wide range of complex CHD which may affect structural brain development and subsequent network topology differently. Our sample size was not large enough to assess differences in network topology related to CHD types. Further studies with larger sample sizes are needed to elucidate whether changes in structural network topology are associated with different CHD types. A common core/periphery structure was defined as nodes belonging to the core/periphery partitioning in 90% of subjects. However, it has been shown that newborns with CHD have fewer rich club nodes compared to controls (De-Asis Cruz et al., 2018). In this case assessing the core/periphery partitioning for the whole group may have influenced our findings. In addition, neurodevelopmental outcome data are not available for this cohort and so we were not able to assess the relationship between our findings and subsequent outcome. However, developmental follow-up of our cohort is currently underway and we will assess this relationship in future studies.

## 5 Conclusion

Using network-based statistics we reveal altered structural connectivity in infants with CHD prior to surgery compared to healthy control infants. We found one subnetwork with reduced structural connectivity in newborns with CHD predominantly affecting core nodes belonging to the cortico-striatal-thalamic network suggesting vulnerability of core connectivity in CHD. Alterations in the sub-network topology of structural connectivity could explain, at least in part, the neurodevelopmental sequelae associated with CHD.

## Funding

This research was funded by the Medical Research Council UK (MR/L011530/1), the British Heart Foundation (FS/15/55/31649), and Action Medical Research (GN2630). This work received funding from the European Research Council under the European Union’s Seventh Framework Program (FP7/20072013)/ERC grant agreement no. 319,456 (dHCP project), and was supported by the Wellcome Engineering and Physical Sciences Research Council Centre for Medical Engineering at Kings College London (WT 203148/Z/16/Z), MRC strategic grant (MR/K006355/1), and by the National Institute for Health Research (NIHR) Biomedical Research Centre based at Guy’s and St Thomas’ NHS Foundation Trust and Kings College London. DB acknowledges support from a Wellcome Trust Seed Award in Science [217316/Z/19/Z]. MNB was supported by the National Children’s Foundation, Tallaght, Ireland. DC is supported by the Flemish Research Foundation (FWO; grant number 12ZV420N). The views expressed are those of the authors and not necessarily those of the NHS, the National Institute for Health Research or the Department of Health. The funders had no role in the design and conduct of the study; collection, management, analysis, and interpretation of the data; preparation, review, or approval of the manuscript; and decision to submit the manuscript for publication.

## CRediT authorship contribution statement

**Megan Ní Bhroin:** Formal analysis, Investigation, Methodology, Visualization, Writing - original draft. **Samy Abo Seada:** Formal analysis, Investigation, Methodology, Writing - review & editing. **Alexandra F. Bonthrone:** Methodology, Resources, Data curation, Writing - review & editing. **Christopher J. Kelly:** Methodology, Resources, Data curation, Writing - review & editing. **Daan Christiaens:** Methodology, Software, Writing - review & editing. **Andreas Schuh:** Methodology, Software, Writing - review & editing. **Maximilian Pietsch:** Methodology, Software, Writing - review & editing. **Jana Hutter:** Methodology, Software, Writing - review & editing. **J-Donald Tournier:** Methodology, Software, Writing - review & editing. **Lucillio Cordero-Grande:** Methodology, Software, Writing - review & editing. **Daniel Rueckert:** Methodology, Software, Writing - review & editing. **Joseph V. Hajnal:** Methodology, Software, Writing - review & editing. **Kuberan Pushparajah:** Validation, Writing - review & editing. **John Simpson:** Validation, Writing - review & editing. **A. David Edwards:** Validation, Writing - review & editing. **Mary A. Rutherford:** Validation, Writing - review & editing. **Serena J. Counsell:** Supervision, Conceptualization, Resources, Methodology, Visualization, Funding acquisition, Project administration, Writing - original draft. **Dafnis Batalle:** Supervision, Conceptualization, Methodology, Resources, Visualization, Writing - original draft.

## Declaration of Competing Interest

The authors declare that they have no known competing financial interests or personal relationships that could have appeared to influence the work reported in this paper.
