# Supplementary material for: Reduced structural connectivity in cortico-striatal-thalamic network in neonates with congenital heart disease
Source: Neuroimage Clin. 2020 Sep 15;28:102423. doi: 10.1016/j.nicl.2020.102423 (PMC7520425; doi:10.1016/j.nicl.2020.102423)
Supplement: Supplementary data 1 [file mmc1.docx]

**Supplementary results 1**

**Supplementary Table 1.** Affected nodes and edges at different *t* thresholds (2.5 to 3.5 in steps of 0.1).

*t* = 2.5

61 nodes, 112 edges, p=0.002

| **#** | **Node** | **Node network** | **#** | **Edge** | **Edge type** | ***t*-value** |
| --- | --- | --- | --- | --- | --- | --- |
| 1 | Precentral gyrus left | Core | 1 | Inferior frontal gyrus (opercular) left to Supplementary motor area right | Peripheral | 2.5 |
| 2 | Precentral gyrus right | Core | 2 | Postcentral gyrus right to Cerebellum right | Feeder | 2.5 |
| 3 | Superior frontal gyrus (dorsal) left | Core | 3 | Thalamus left to Vermis | Core | 2.5 |
| 4 | Orbitofrontal cortex (superior) left | Peripheral | 4 | Olfactory right to Rectus gyrus right | Peripheral | 2.51 |
| 5 | Middle frontal gyrus left | Core | 5 | Thalamus right to Cerebellum right | Core | 2.51 |
| 6 | Middle frontal gyrus right | Core | 6 | Angular gyrus right to Vermis | Core | 2.51 |
| 7 | Orbitofrontal cortex (medial) left | Peripheral | 7 | Superior frontal gyrus (dorsal) left to Hippocampus left | Feeder | 2.52 |
| 8 | Orbitofrontal cortex (medial) right | Peripheral | 8 | Hippocampus left to Thalamus left | Feeder | 2.53 |
| 9 | Inferior frontal gyrus (opercular) left | Peripheral | 9 | Precentral gyrus left to Postcentral gyrus right | Feeder | 2.54 |
| 10 | Inferior frontal gyrus (opercular) right | Peripheral | 10 | Rectus gyrus left to Inferior occipital gyrus left | Peripheral | 2.55 |
| 11 | Inferior frontal gyrus (triangular) left | Core | 11 | Orbitofrontal cortex (medial) left to Orbitofrontal cortex (inferior) left | Peripheral | 2.56 |
| 12 | Inferior frontal gyrus (triangular) right | Peripheral | 12 | Orbitofrontal cortex (medial) left to Posterior cingulate gyrus right | Peripheral | 2.56 |
| 13 | Orbitofrontal cortex (inferior) left | Peripheral | 13 | Median cingulate and paracingulate gyrus left to Hippocampus left | Feeder | 2.57 |
| 14 | Rolandic operculum left | Peripheral | 14 | Inferior frontal gyrus (triangular) left to Hippocampus left | Feeder | 2.58 |
| 15 | Supplementary motor area left | Peripheral | 15 | Rectus gyrus left to Superior temporal gyrus left | Peripheral | 2.58 |
| 16 | Supplementary motor area right | Peripheral | 16 | Angular gyrus right to Cerebellum left | Core | 2.59 |
| 17 | Olfactory right | Peripheral | 17 | Thalamus left to Cerebellum right | Core | 2.6 |
| 18 | Superior frontal gyrus (medial) left | Core | 18 | Inferior frontal gyrus (triangular) left to Postcentral gyrus right | Feeder | 2.61 |
| 19 | Rectus gyrus left | Peripheral | 19 | Postcentral gyrus right to Temporal pole (superior) left | Peripheral | 2.61 |
| 20 | Rectus gyrus right | Peripheral | 20 | Angular gyrus left to Cerebellum right | Feeder | 2.61 |
| 21 | Insula left | Core | 21 | Olfactory right to Caudate right | Feeder | 2.63 |
| 22 | Anterior cingulate gyrus left | Core | 22 | Rectus gyrus left to Insula left | Feeder | 2.64 |
| 23 | Median cingulate and paracingulate gyrus left | Core | 23 | Rectus gyrus left to Angular gyrus left | Peripheral | 2.65 |
| 24 | Posterior cingulate gyrus right | Peripheral | 24 | Precentral gyrus left to Supramarginal gyrus left | Feeder | 2.66 |
| 25 | Hippocampus left | Peripheral | 25 | Hippocampus left to Caudate left | Feeder | 2.66 |
| 26 | Hippocampus right | Peripheral | 26 | Middle frontal gyrus right to Anterior cingulate gyrus left | Core | 2.68 |
| 27 | Amygdala left | Peripheral | 27 | Hippocampus right to Cerebellum left | Feeder | 2.68 |
| 28 | Amygdala right | Peripheral | 28 | Middle frontal gyrus right to Vermis | Core | 2.71 |
| 29 | Cuneus right | Peripheral | 29 | Superior frontal gyrus (dorsal) left to Postcentral gyrus right | Feeder | 2.72 |
| 30 | Superior occipital gyrus left | Peripheral | 30 | Supplementary motor area left to Supramarginal gyrus left | Peripheral | 2.72 |
| 31 | Superior occipital gyrus right | Peripheral | 31 | Middle frontal gyrus right to Orbitofrontal cortex (medial) right | Feeder | 2.73 |
| 32 | Middle occipital gyrus left | Core | 32 | Rolandic operculum left to Rectus gyrus left | Peripheral | 2.73 |
| 33 | Middle occipital gyrus right | Peripheral | 33 | Middle frontal gyrus right to Postcentral gyrus right | Feeder | 2.73 |
| 34 | Inferior occipital gyrus left | Peripheral | 34 | Inferior frontal gyrus (opercular) right to Hippocampus right | Peripheral | 2.74 |
| 35 | Postcentral gyrus left | Peripheral | 35 | Superior frontal gyrus (dorsal) left to Supramarginal gyrus left | Feeder | 2.74 |
| 36 | Postcentral gyrus right | Peripheral | 36 | Posterior cingulate gyrus right to Precuneus right | Feeder | 2.74 |
| 37 | Superior parietal gyrus left | Peripheral | 37 | Postcentral gyrus right to Supramarginal gyrus left | Peripheral | 2.75 |
| 38 | Inferior parietal lobule left | Core | 38 | Superior occipital gyrus left to Paracentral lobule right | Peripheral | 2.76 |
| 39 | Inferior parietal lobule right | Peripheral | 39 | Inferior frontal gyrus (triangular) right to Hippocampus right | Peripheral | 2.77 |
| 40 | Supramarginal gyrus left | Peripheral | 40 | Hippocampus right to Thalamus right | Feeder | 2.77 |
| 41 | Supramarginal gyrus right | Peripheral | 41 | Middle occipital gyrus left to Cerebellum right | Core | 2.77 |
| 42 | Angular gyrus left | Peripheral | 42 | Postcentral gyrus right to Putamen left | Feeder | 2.79 |
| 43 | Angular gyrus right | Core | 43 | Precentral gyrus left to Cerebellum right | Core | 2.79 |
| 44 | Precuneus left | Core | 44 | Inferior frontal gyrus (opercular) right to Postcentral gyrus right | Peripheral | 2.8 |
| 45 | Precuneus right | Core | 45 | Hippocampus right to Cerebellum right | Feeder | 2.82 |
| 46 | Paracentral lobule right | Peripheral | 46 | Rolandic operculum left to Supplementary motor area right | Peripheral | 2.83 |
| 47 | Caudate left | Core | 47 | Middle frontal gyrus right to Hippocampus right | Feeder | 2.83 |
| 48 | Caudate right | Core | 48 | Caudate left to Caudate right | Core | 2.83 |
| 49 | Putamen left | Core | 49 | Supplementary motor area left to Cerebellum right | Feeder | 2.83 |
| 50 | Putamen right | Core | 50 | Orbitofrontal cortex (medial) right to Caudate right | Feeder | 2.84 |
| 51 | Thalamus left | Core | 51 | Inferior parietal lobule right to Vermis | Feeder | 2.84 |
| 52 | Thalamus right | Core | 52 | Middle frontal gyrus right to Inferior frontal gyrus (triangular) left | Core | 2.85 |
| 53 | Heschl gyrus left | Peripheral | 53 | Hippocampus left to Postcentral gyrus left | Peripheral | 2.85 |
| 54 | Superior temporal gyrus left | Peripheral | 54 | Hippocampus left to Inferior parietal lobule left | Feeder | 2.85 |
| 55 | Temporal pole (superior) left | Peripheral | 55 | Precentral gyrus left to Precuneus left | Core | 2.85 |
| 56 | Middle temporal gyrus left | Core | 56 | Supplementary motor area left to Hippocampus left | Peripheral | 2.86 |
| 57 | Inferior temporal gyrus left | Peripheral | 57 | Precentral gyrus right to Cerebellum right | Core | 2.86 |
| 58 | Inferior temporal gyrus right | Core | 58 | Middle frontal gyrus right to Orbitofrontal cortex (medial) left | Feeder | 2.87 |
| 59 | Cerebellum left | Core | 59 | Hippocampus left to Superior occipital gyrus left | Peripheral | 2.87 |
| 60 | Cerebellum right | Core | 60 | Amygdala right to Inferior parietal lobule right | Peripheral | 2.89 |
| 61 | Vermis | Core | 61 | Putamen left to Vermis | Core | 2.9 |
|  |  |  | 62 | Rolandic operculum left to Supplementary motor area left | Peripheral | 2.91 |
|  |  |  | 63 | Superior frontal gyrus (dorsal) left to Middle frontal gyrus right | Core | 2.92 |
|  |  |  | 64 | Amygdala right to Angular gyrus right | Feeder | 2.92 |
|  |  |  | 65 | Orbitofrontal cortex (superior) left to Middle frontal gyrus right | Feeder | 2.97 |
|  |  |  | 66 | Inferior frontal gyrus (triangular) right to Postcentral gyrus right | Peripheral | 2.98 |
|  |  |  | 67 | Postcentral gyrus right to Cerebellum left | Feeder | 2.98 |
|  |  |  | 68 | Cuneus right to Middle occipital gyrus right | Peripheral | 2.99 |
|  |  |  | 69 | Hippocampus right to Superior occipital gyrus right | Peripheral | 3 |
|  |  |  | 70 | Superior occipital gyrus left to Postcentral gyrus right | Peripheral | 3 |
|  |  |  | 71 | Thalamus right to Vermis | Core | 3 |
|  |  |  | 72 | Inferior frontal gyrus (opercular) left to Postcentral gyrus right | Peripheral | 3.02 |
|  |  |  | 73 | Hippocampus left to Angular gyrus left | Peripheral | 3.05 |
|  |  |  | 74 | Rectus gyrus left to Heschl gyrus left | Peripheral | 3.05 |
|  |  |  | 75 | Supplementary motor area left to Postcentral gyrus right | Peripheral | 3.06 |
|  |  |  | 76 | Hippocampus right to Putamen right | Feeder | 3.06 |
|  |  |  | 77 | Hippocampus right to Supramarginal gyrus right | Peripheral | 3.07 |
|  |  |  | 78 | Middle frontal gyrus right to Caudate right | Core | 3.07 |
|  |  |  | 79 | Median cingulate and paracingulate gyrus left to Postcentral gyrus right | Feeder | 3.08 |
|  |  |  | 80 | Hippocampus left to Middle occipital gyrus left | Feeder | 3.11 |
|  |  |  | 81 | Precentral gyrus right to Hippocampus right | Feeder | 3.12 |
|  |  |  | 82 | Caudate right to Inferior temporal gyrus right | Core | 3.13 |
|  |  |  | 83 | Precentral gyrus right to Cerebellum left | Core | 3.13 |
|  |  |  | 84 | Middle frontal gyrus left to Middle frontal gyrus right | Core | 3.14 |
|  |  |  | 85 | Orbitofrontal cortex (superior) left to Orbitofrontal cortex (medial) left | Peripheral | 3.14 |
|  |  |  | 86 | Middle occipital gyrus left to Middle temporal gyrus left | Core | 3.15 |
|  |  |  | 87 | Middle occipital gyrus left to Inferior occipital gyrus left | Feeder | 3.17 |
|  |  |  | 88 | Hippocampus left to Superior parietal gyrus left | Peripheral | 3.17 |
|  |  |  | 89 | Inferior occipital gyrus left to Middle temporal gyrus left | Feeder | 3.29 |
|  |  |  | 90 | Hippocampus right to Inferior parietal lobule right | Peripheral | 3.31 |
|  |  |  | 91 | Hippocampus left to Amygdala left | Peripheral | 3.34 |
|  |  |  | 92 | Hippocampus left to Cerebellum left | Feeder | 3.36 |
|  |  |  | 93 | Hippocampus right to Middle occipital gyrus right | Peripheral | 3.4 |
|  |  |  | 94 | Postcentral gyrus left to Cerebellum right | Feeder | 3.43 |
|  |  |  | 95 | Inferior frontal gyrus (triangular) left to Rolandic operculum left | Feeder | 3.44 |
|  |  |  | 96 | Inferior frontal gyrus (opercular) left to Inferior frontal gyrus (triangular) left | Feeder | 3.46 |
|  |  |  | 97 | Rectus gyrus left to Supramarginal gyrus left | Peripheral | 3.47 |
|  |  |  | 98 | Rolandic operculum left to Superior frontal gyrus (medial) left | Feeder | 3.49 |
|  |  |  | 99 | Thalamus right to Cerebellum left | Core | 3.51 |
|  |  |  | 100 | Putamen right to Thalamus right | Core | 3.52 |
|  |  |  | 101 | Thalamus right to Inferior temporal gyrus right | Core | 3.52 |
|  |  |  | 102 | Hippocampus right to Angular gyrus right | Feeder | 3.58 |
|  |  |  | 103 | Precentral gyrus right to Vermis | Core | 3.7 |
|  |  |  | 104 | Superior frontal gyrus (dorsal) left to Rolandic operculum left | Feeder | 3.71 |
|  |  |  | 105 | Inferior occipital gyrus left to Inferior temporal gyrus left | Peripheral | 3.72 |
|  |  |  | 106 | Hippocampus right to Postcentral gyrus right | Peripheral | 3.77 |
|  |  |  | 107 | Putamen left to Thalamus left | Core | 3.77 |
|  |  |  | 108 | Caudate right to Putamen left | Core | 3.81 |
|  |  |  | 109 | Cerebellum left to Cerebellum right | Core | 3.81 |
|  |  |  | 110 | Hippocampus left to Vermis | Feeder | 3.93 |
|  |  |  | 111 | Hippocampus left to Cerebellum right | Feeder | 3.97 |
|  |  |  | 112 | Hippocampus right to Caudate right | Feeder | 4 |

*t* = 2.6

56 nodes, 95 edges, p=0.001

| **#** | **Node** | **Node network** | **#** | **Edge** | **Edge type** | ***t*-value** |
| --- | --- | --- | --- | --- | --- | --- |
| 1 | Precentral gyrus left | Core | 1 | Thalamus left to Cerebellum right | Core | 2.6 |
| 2 | Precentral gyrus right | Core | 2 | Inferior frontal gyrus (triangular) left to Postcentral gyrus right | Feeder | 2.61 |
| 3 | Superior frontal gyrus (dorsal) left | Core | 3 | Postcentral gyrus right to Temporal pole (superior) left | Peripheral | 2.61 |
| 4 | Orbitofrontal cortex (superior) left | Peripheral | 4 | Angular gyrus left to Cerebellum right | Feeder | 2.61 |
| 5 | Middle frontal gyrus left | Core | 5 | Olfactory right to Caudate right | Feeder | 2.63 |
| 6 | Middle frontal gyrus right | Core | 6 | Rectus gyrus left to Insula left | Feeder | 2.64 |
| 7 | Orbitofrontal cortex (medial) left | Peripheral | 7 | Rectus gyrus left to Angular gyrus left | Peripheral | 2.65 |
| 8 | Orbitofrontal cortex (medial) right | Peripheral | 8 | Precentral gyrus left to Supramarginal gyrus left | Feeder | 2.66 |
| 9 | Inferior frontal gyrus (opercular) left | Peripheral | 9 | Hippocampus left to Caudate right | Feeder | 2.66 |
| 10 | Inferior frontal gyrus (opercular) right | Peripheral | 10 | Middle frontal gyrus right to Anterior cingulate gyrus left | Core | 2.68 |
| 11 | Inferior frontal gyrus (triangular) left | Core | 11 | Hippocampus right to Cerebellum left | Feeder | 2.68 |
| 12 | Inferior frontal gyrus (triangular) right | Peripheral | 12 | Middle frontal gyrus right to Vermis | Core | 2.71 |
| 13 | Rolandic operculum left | Peripheral | 13 | Superior frontal gyrus (dorsal) left to Postcentral gyrus left | Feeder | 2.72 |
| 14 | Supplementary motor area left | Peripheral | 14 | Supplementary motor area left to Supramarginal gyrus left | Peripheral | 2.72 |
| 15 | Supplementary motor area right | Peripheral | 15 | Middle frontal gyrus right to Orbitofrontal cortex (medial) right | Feeder | 2.73 |
| 16 | Olfactory right | Peripheral | 16 | Rolandic operculum left to Rectus gyrus left | Peripheral | 2.73 |
| 17 | Superior frontal gyrus (medial) left | Core | 17 | Middle frontal gyrus right to Postcentral gyrus left | Feeder | 2.73 |
| 18 | Rectus gyrus left | Peripheral | 18 | Inferior frontal gyrus (opercular) right to Hippocampus right | Peripheral | 2.74 |
| 19 | Insula left | Core | 19 | Superior frontal gyrus (dorsal) left to Supramarginal gyrus left | Feeder | 2.74 |
| 20 | Anterior cingulate gyrus left | Core | 20 | Postcentral gyrus right to Supramarginal gyrus left | Peripheral | 2.75 |
| 21 | Median cingulate and paracingulate gyrus left | Core | 21 | Superior occipital gyrus left to Paracentral lobule right | Peripheral | 2.76 |
| 22 | Hippocampus left | Peripheral | 22 | Inferior frontal gyrus (triangular) right to Hippocampus right | Peripheral | 2.77 |
| 23 | Hippocampus right | Peripheral | 23 | Hippocampus right to Thalamus right | Feeder | 2.77 |
| 24 | Amygdala left | Peripheral | 24 | Middle occipital gyrus left to Cerebellum right | Core | 2.77 |
| 25 | Amygdala right | Peripheral | 25 | Postcentral gyrus right to Putamen left | Feeder | 2.79 |
| 26 | Cuneus right | Peripheral | 26 | Precentral gyrus left to Cerebellum right | Core | 2.79 |
| 27 | Superior occipital gyrus left | Peripheral | 27 | Inferior frontal gyrus (opercular) right to Postcentral gyrus right | Peripheral | 2.8 |
| 28 | Superior occipital gyrus right | Peripheral | 28 | Hippocampus right to Cerebellum right | Feeder | 2.82 |
| 29 | Middle occipital gyrus left | Core | 29 | Rolandic operculum left to Supplementary motor area right | Peripheral | 2.83 |
| 30 | Middle occipital gyrus right | Peripheral | 30 | Middle frontal gyrus right to Hippocampus right | Feeder | 2.83 |
| 31 | Inferior occipital gyrus left | Peripheral | 31 | Caudate left to Caudate right | Core | 2.83 |
| 32 | Postcentral gyrus left | Peripheral | 32 | Supplementary motor area left to Cerebellum right | Feeder | 2.83 |
| 33 | Postcentral gyrus right | Peripheral | 33 | Orbitofrontal cortex (medial) right to Caudate right | Feeder | 2.84 |
| 34 | Superior parietal gyrus left | Peripheral | 34 | Inferior parietal lobule right to Vermis | Feeder | 2.84 |
| 35 | Inferior parietal lobule left | Core | 35 | Middle frontal gyrus right to Inferior frontal gyrus (triangular) left | Core | 2.85 |
| 36 | Inferior parietal lobule right | Peripheral | 36 | Hippocampus left to Postcentral gyrus left | Peripheral | 2.85 |
| 37 | Supramarginal gyrus left | Peripheral | 37 | Hippocampus left to Inferior parietal lobule left | Feeder | 2.85 |
| 38 | Supramarginal gyrus right | Peripheral | 38 | Precentral gyrus left to Precuneus left | Core | 2.85 |
| 39 | Angular gyrus left | Peripheral | 39 | Supplementary motor area left to Hippocampus left | Peripheral | 2.86 |
| 40 | Angular gyrus right | Core | 40 | Precentral gyrus right to Cerebellum right | Core | 2.86 |
| 41 | Precuneus left | Core | 41 | Middle frontal gyrus right to Orbitofrontal cortex (medial) left | Feeder | 2.87 |
| 42 | Paracentral lobule right | Peripheral | 42 | Hippocampus left to Superior occipital gyrus left | Peripheral | 2.87 |
| 43 | Caudate left | Core | 43 | Amygdala right to Inferior parietal lobule right | Peripheral | 2.89 |
| 44 | Caudate right | Core | 44 | Putamen left to Vermis | Core | 2.9 |
| 45 | Putamen left | Core | 45 | Rolandic operculum left to Supplementary motor area left | Peripheral | 2.91 |
| 46 | Putamen right | Core | 46 | Superior frontal gyrus (dorsal) left to Middle frontal gyrus right | Core | 2.92 |
| 47 | Thalamus left | Core | 47 | Amygdala right to Angular gyrus right | Feeder | 2.92 |
| 48 | Thalamus right | Core | 48 | Orbitofrontal cortex (superior) left to Middle frontal gyrus right | Feeder | 2.97 |
| 49 | Heschl gyrus left | Peripheral | 49 | Inferior frontal gyrus (triangular) right to Postcentral gyrus right | Peripheral | 2.98 |
| 50 | Temporal pole (superior) left | Peripheral | 50 | Postcentral gyrus right to Cerebellum left | Feeder | 2.98 |
| 51 | Middle temporal gyrus left | Core | 51 | Cuneus right to Middle occipital gyrus right | Peripheral | 2.99 |
| 52 | Inferior temporal gyrus left | Peripheral | 52 | Hippocampus right to Superior occipital gyrus right | Peripheral | 3 |
| 53 | Inferior temporal gyrus right | Core | 53 | Superior occipital gyrus left to Postcentral gyrus left | Peripheral | 3 |
| 54 | Cerebellum left | Core | 54 | Thalamus right to Vermis | Core | 3 |
| 55 | Cerebellum right | Core | 55 | Inferior frontal gyrus (opercular) left to Postcentral gyrus right | Peripheral | 3.02 |
| 56 | Vermis | Core | 56 | Hippocampus left to Angular gyrus left | Peripheral | 3.05 |
|  |  |  | 57 | Rectus gyrus left to Heschl gyrus left | Peripheral | 3.05 |
|  |  |  | 58 | Supplementary motor area left to Postcentral gyrus left | Peripheral | 3.06 |
|  |  |  | 59 | Hippocampus right to Putamen right | Feeder | 3.06 |
|  |  |  | 60 | Hippocampus right to Supramarginal gyrus right | Peripheral | 3.07 |
|  |  |  | 61 | Middle frontal gyrus right to Caudate right | Core | 3.07 |
|  |  |  | 62 | Median cingulate and paracingulate gyrus left to Postcentral gyrus left | Feeder | 3.08 |
|  |  |  | 63 | Hippocampus left to Middle occipital gyrus left | Feeder | 3.11 |
|  |  |  | 64 | Precentral gyrus right to Hippocampus right | Feeder | 3.12 |
|  |  |  | 65 | Caudate right to Inferior temporal gyrus right | Core | 3.13 |
|  |  |  | 66 | Precentral gyrus right to Cerebellum left | Core | 3.13 |
|  |  |  | 67 | Middle frontal gyrus left to Middle frontal gyrus right | Core | 3.14 |
|  |  |  | 68 | Orbitofrontal cortex (superior) left to Orbitofrontal cortex (medial) left | Peripheral | 3.14 |
|  |  |  | 69 | Middle occipital gyrus left to Middle temporal gyrus left | Core | 3.15 |
|  |  |  | 70 | Middle occipital gyrus left to Inferior occipital gyrus left | Feeder | 3.17 |
|  |  |  | 71 | Hippocampus left to Superior parietal gyrus left | Peripheral | 3.17 |
|  |  |  | 72 | Inferior occipital gyrus left to Middle temporal gyrus left | Feeder | 3.29 |
|  |  |  | 73 | Hippocampus right to Inferior parietal lobule right | Peripheral | 3.31 |
|  |  |  | 74 | Hippocampus left to Amygdala left | Peripheral | 3.34 |
|  |  |  | 75 | Hippocampus left to Cerebellum left | Feeder | 3.36 |
|  |  |  | 76 | Hippocampus right to Middle occipital gyrus right | Peripheral | 3.4 |
|  |  |  | 77 | Postcentral gyrus left to Cerebellum right | Feeder | 3.43 |
|  |  |  | 78 | Inferior frontal gyrus (triangular) left to Rolandic operculum left | Feeder | 3.44 |
|  |  |  | 79 | Inferior frontal gyrus (opercular) left to Inferior frontal gyrus (triangular) left | Feeder | 3.46 |
|  |  |  | 80 | Rectus gyrus left to Supramarginal gyrus left | Peripheral | 3.47 |
|  |  |  | 81 | Rolandic operculum left to Superior frontal gyrus (medial) left | Feeder | 3.49 |
|  |  |  | 82 | Thalamus right to Cerebellum left | Core | 3.51 |
|  |  |  | 83 | Putamen right to Thalamus right | Core | 3.52 |
|  |  |  | 84 | Thalamus right to Inferior temporal gyrus right | Core | 3.52 |
|  |  |  | 85 | Hippocampus right to Angular gyrus right | Feeder | 3.58 |
|  |  |  | 86 | Precentral gyrus right to Vermis | Core | 3.7 |
|  |  |  | 87 | Superior frontal gyrus (dorsal) left to Rolandic operculum left | Feeder | 3.71 |
|  |  |  | 88 | Inferior occipital gyrus left to Inferior temporal gyrus left | Peripheral | 3.72 |
|  |  |  | 89 | Hippocampus right to Postcentral gyrus left | Peripheral | 3.77 |
|  |  |  | 90 | Putamen left to Thalamus left | Core | 3.77 |
|  |  |  | 91 | Caudate right to Putamen left | Core | 3.81 |
|  |  |  | 92 | Cerebellum left to Cerebellum right | Core | 3.81 |
|  |  |  | 93 | Hippocampus left to Vermis | Feeder | 3.93 |
|  |  |  | 94 | Hippocampus left to Cerebellum right | Feeder | 3.97 |
|  |  |  | 95 | Hippocampus right to Caudate right | Feeder | 4 |

*t =* 2.7

52 nodes, 84 edges, p=0.05

| **#** | **Node** | **Node network** | **#** | **Edge** | **Edge type** | ***t*-value** |
| --- | --- | --- | --- | --- | --- | --- |
| 1 | Precentral gyrus left | Core | 1 | Middle frontal gyrus right to Vermis | Core | 2.71 |
| 2 | Precentral gyrus right | Core | 2 | Superior frontal gyrus (dorsal) left to Postcentral gyrus right | Feeder | 2.72 |
| 3 | Superior frontal gyrus (dorsal) left | Core | 3 | Supplementary motor area left to Supramarginal gyrus left | Peripheral | 2.72 |
| 4 | Orbitofrontal cortex (superior) left | Periphery | 4 | Middle frontal gyrus right to Orbitofrontal cortex (medial) right | Feeder | 2.73 |
| 5 | Middle frontal gyrus left | Core | 5 | Rolandic operculum left to Rectus gyrus left | Peripheral | 2.73 |
| 6 | Middle frontal gyrus right | Core | 6 | Middle frontal gyrus right to Postcentral gyrus right | Feeder | 2.73 |
| 7 | Orbitofrontal cortex (medial) left | Periphery | 7 | Inferior frontal gyrus (opercular) right to Hippocampus right | Peripheral | 2.74 |
| 8 | Orbitofrontal cortex (medial) right | Periphery | 8 | Superior frontal gyrus (dorsal) left to Supramarginal gyrus left | Feeder | 2.74 |
| 9 | Inferior frontal gyrus (opercular) left | Periphery | 9 | Postcentral gyrus right to Supramarginal gyrus left | Peripheral | 2.75 |
| 10 | Inferior frontal gyrus (opercular) right | Periphery | 10 | Superior occipital gyrus left to Paracentral lobule right | Peripheral | 2.76 |
| 11 | Inferior frontal gyrus (triangular) left | Core | 11 | Inferior frontal gyrus (triangular) right to Hippocampus right | Peripheral | 2.77 |
| 12 | Inferior frontal gyrus (triangular) right | Periphery | 12 | Hippocampus right to Thalamus right | Feeder | 2.77 |
| 13 | Rolandic operculum left | Periphery | 13 | Middle occipital gyrus left to Cerebellum right | Core | 2.77 |
| 14 | Supplementary motor area left | Peripheral | 14 | Postcentral gyrus right to Putamen left | Feeder | 2.79 |
| 15 | Supplementary motor area right | Peripheral | 15 | Precentral gyrus left to Cerebellum right | Core | 2.79 |
| 16 | Superior frontal gyrus (medial) left | Core | 16 | Inferior frontal gyrus (opercular) right to Postcentral gyrus right | Peripheral | 2.8 |
| 17 | Rectus gyrus left | Peripheral | 17 | Hippocampus right to Cerebellum right | Feeder | 2.82 |
| 18 | Median cingulate and paracingulate gyrus left | Core | 18 | Rolandic operculum left to Supplementary motor area right | Peripheral | 2.83 |
| 19 | Hippocampus left | Peripheral | 19 | Middle frontal gyrus right to Hippocampus right | Feeder | 2.83 |
| 20 | Hippocampus right | Peripheral | 20 | Caudate left to Caudate right | Core | 2.83 |
| 21 | Amygdala left | Peripheral | 21 | Supplementary motor area left to Cerebellum right | Feeder | 2.83 |
| 22 | Amygdala right | Peripheral | 22 | Orbitofrontal cortex (medial) right to Caudate right | Feeder | 2.84 |
| 23 | Cuneus right | Peripheral | 23 | Inferior parietal lobule right to Vermis | Feeder | 2.84 |
| 24 | Superior occipital gyrus left | Peripheral | 24 | Middle frontal gyrus right to Inferior frontal gyrus (triangular) left | Core | 2.85 |
| 25 | Superior occipital gyrus right | Peripheral | 25 | Hippocampus left to Postcentral gyrus left | Peripheral | 2.85 |
| 26 | Middle occipital gyrus left | Core | 26 | Hippocampus left to Inferior parietal lobule left | Feeder | 2.85 |
| 27 | Middle occipital gyrus right | Peripheral | 27 | Precentral gyrus left to Precuneus left | Core | 2.85 |
| 28 | Inferior occipital gyrus left | Peripheral | 28 | Supplementary motor area left to Hippocampus left | Peripheral | 2.86 |
| 29 | Postcentral gyrus left | Peripheral | 29 | Precentral gyrus right to Cerebellum right | Core | 2.86 |
| 30 | Postcentral gyrus right | Peripheral | 30 | Middle frontal gyrus right to Orbitofrontal cortex (medial) left | Feeder | 2.87 |
| 31 | Superior parietal gyrus left | Peripheral | 31 | Hippocampus left to Superior occipital gyrus left | Peripheral | 2.87 |
| 32 | Inferior parietal lobule left | Core | 32 | Amygdala right to Inferior parietal lobule right | Peripheral | 2.89 |
| 33 | Inferior parietal lobule right | Peripheral | 33 | Putamen left to Vermis | Core | 2.9 |
| 34 | Supramarginal gyrus left | Peripheral | 34 | Rolandic operculum left to Supplementary motor area left | Peripheral | 2.91 |
| 35 | Supramarginal gyrus right | Peripheral | 35 | Superior frontal gyrus (dorsal) left to Middle frontal gyrus right | Core | 2.92 |
| 36 | Angular gyrus left | Peripheral | 36 | Amygdala right to Angular gyrus right | Feeder | 2.92 |
| 37 | Angular gyrus right | Core | 37 | Orbitofrontal cortex (superior) left to Middle frontal gyrus right | Feeder | 2.97 |
| 38 | Precuneus left | Core | 38 | Inferior frontal gyrus (triangular) right to Postcentral gyrus right | Peripheral | 2.98 |
| 39 | Paracentral lobule right | Peripheral | 39 | Postcentral gyrus right to Cerebellum left | Feeder | 2.98 |
| 40 | Caudate left | Core | 40 | Cuneus right to Middle occipital gyrus right | Peripheral | 2.99 |
| 41 | Caudate right | Core | 41 | Hippocampus right to Superior occipital gyrus right | Peripheral | 3 |
| 42 | Putamen left | Core | 42 | Superior occipital gyrus left to Postcentral gyrus right | Peripheral | 3 |
| 43 | Putamen right | Core | 43 | Thalamus right to Vermis | Core | 3 |
| 44 | Thalamus left | Core | 44 | Inferior frontal gyrus (opercular) left to Postcentral gyrus right | Peripheral | 3.02 |
| 45 | Thalamus right | Core | 45 | Hippocampus left to Angular gyrus left | Peripheral | 3.05 |
| 46 | Heschl gyrus left | Peripheral | 46 | Rectus gyrus left to Heschl gyrus left | Peripheral | 3.05 |
| 47 | Middle temporal gyrus left | Core | 47 | Supplementary motor area left to Postcentral gyrus right | Peripheral | 3.06 |
| 48 | Inferior temporal gyrus left | Peripheral | 48 | Hippocampus right to Putamen right | Feeder | 3.06 |
| 49 | Inferior temporal gyrus right | Core | 49 | Hippocampus right to Supramarginal gyrus right | Peripheral | 3.07 |
| 50 | Cerebellum left | Core | 50 | Middle frontal gyrus right to Caudate right | Core | 3.07 |
| 51 | Cerebellum right | Core | 51 | Median cingulate and paracingulate gyrus left to Postcentral gyrus right | Feeder | 3.08 |
| 52 | Vermis | Core | 52 | Hippocampus left to Middle occipital gyrus left | Feeder | 3.11 |
|  |  |  | 53 | Precentral gyrus right to Hippocampus right | Feeder | 3.12 |
|  |  |  | 54 | Caudate right to Inferior temporal gyrus right | Core | 3.13 |
|  |  |  | 55 | Precentral gyrus right to Cerebellum left | Core | 3.13 |
|  |  |  | 56 | Middle frontal gyrus left to Middle frontal gyrus right | Core | 3.14 |
|  |  |  | 57 | Orbitofrontal cortex (superior) left to Orbitofrontal cortex (medial) left | Peripheral | 3.14 |
|  |  |  | 58 | Middle occipital gyrus left to Middle temporal gyrus left | Core | 3.15 |
|  |  |  | 59 | Middle occipital gyrus left to Inferior occipital gyrus left | Feeder | 3.17 |
|  |  |  | 60 | Hippocampus left to Superior parietal gyrus left | Peripheral | 3.17 |
|  |  |  | 61 | Inferior occipital gyrus left to Middle temporal gyrus left | Feeder | 3.29 |
|  |  |  | 62 | Hippocampus right to Inferior parietal lobule right | Peripheral | 3.31 |
|  |  |  | 63 | Hippocampus left to Amygdala left | Peripheral | 3.34 |
|  |  |  | 64 | Hippocampus left to Cerebellum left | Feeder | 3.36 |
|  |  |  | 65 | Hippocampus right to Middle occipital gyrus right | Peripheral | 3.4 |
|  |  |  | 66 | Postcentral gyrus left to Cerebellum right | Feeder | 3.43 |
|  |  |  | 67 | Inferior frontal gyrus (triangular) left to Rolandic operculum left | Feeder | 3.44 |
|  |  |  | 68 | Inferior frontal gyrus (opercular) left to Inferior frontal gyrus (triangular) left | Feeder | 3.46 |
|  |  |  | 69 | Rectus gyrus left to Supramarginal gyrus left | Peripheral | 3.47 |
|  |  |  | 70 | Rolandic operculum left to Superior frontal gyrus (medial) left | Feeder | 3.49 |
|  |  |  | 71 | Thalamus right to Cerebellum left | Core | 3.51 |
|  |  |  | 72 | Putamen right to Thalamus right | Core | 3.52 |
|  |  |  | 73 | Thalamus right to Inferior temporal gyrus right | Core | 3.52 |
|  |  |  | 74 | Hippocampus right to Angular gyrus right | Feeder | 3.58 |
|  |  |  | 75 | Precentral gyrus right to Vermis | Core | 3.7 |
|  |  |  | 76 | Superior frontal gyrus (dorsal) left to Rolandic operculum left | Feeder | 3.71 |
|  |  |  | 77 | Inferior occipital gyrus left to Inferior temporal gyrus left | Peripheral | 3.72 |
|  |  |  | 78 | Hippocampus right to Postcentral gyrus right | Peripheral | 3.77 |
|  |  |  | 79 | Putamen left to Thalamus left | Core | 3.77 |
|  |  |  | 80 | Caudate right to Putamen left | Core | 3.81 |
|  |  |  | 81 | Cerebellum left to Cerebellum right | Core | 3.81 |
|  |  |  | 82 | Hippocampus left to Vermis | Feeder | 3.93 |
|  |  |  | 83 | Hippocampus left to Cerebellum right | Feeder | 3.97 |
|  |  |  | 84 | Hippocampus right to Caudate right | Feeder | 4 |

*t =* 2.8

46 nodes, 66 edges, p<0.001

| **#** | **Node** | **Node network** | **#** | **Edge** | **Edge type** | ***t*-value** |
| --- | --- | --- | --- | --- | --- | --- |
| 1 | Precentral gyrus right | Core | 1 | Inferior frontal gyrus (opercular) right to Postcentral gyrus right | Peripheral | 2.8 |
| 2 | Superior frontal gyrus (dorsal) left | Core | 2 | Hippocampus right to Cerebellum right | Feeder | 2.82 |
| 3 | Orbitofrontal cortex (superior) left | Peripheral | 3 | Rolandic operculum left to Supplementary motor area right | Peripheral | 2.83 |
| 4 | Middle frontal gyrus left | Core | 4 | Middle frontal gyrus right to Hippocampus right | Feeder | 2.83 |
| 5 | Middle frontal gyrus right | Core | 5 | Caudate left to Caudate right | Core | 2.83 |
| 6 | Orbitofrontal cortex (medial) left | Peripheral | 6 | Supplementary motor area left to Cerebellum right | Feeder | 2.83 |
| 7 | Orbitofrontal cortex (medial) right | Peripheral | 7 | Orbitofrontal cortex (medial) right to Caudate right | Feeder | 2.84 |
| 8 | Inferior frontal gyrus (opercular) left | Peripheral | 8 | Inferior parietal lobule right to Vermis | Feeder | 2.84 |
| 9 | Inferior frontal gyrus (opercular) right | Peripheral | 9 | Middle frontal gyrus right to Inferior frontal gyrus (triangular) left | Core | 2.85 |
| 10 | Inferior frontal gyrus (triangular) left | Core | 10 | Hippocampus left to Postcentral gyrus left | Peripheral | 2.85 |
| 11 | Inferior frontal gyrus (triangular) right | Peripheral | 11 | Hippocampus left to Inferior parietal lobule left | Feeder | 2.85 |
| 12 | Rolandic operculum left | Peripheral | 12 | Supplementary motor area left to Hippocampus left | Peripheral | 2.86 |
| 13 | Supplementary motor area left | Peripheral | 13 | Precentral gyrus right to Cerebellum right | Core | 2.86 |
| 14 | Supplementary motor area right | Peripheral | 14 | Middle frontal gyrus right to Orbitofrontal cortex (medial) left | Feeder | 2.87 |
| 15 | Superior frontal gyrus (medial) left | Core | 15 | Hippocampus left to Superior occipital gyrus left | Peripheral | 2.87 |
| 16 | Median cingulate and paracingulate gyrus left | Core | 16 | Amygdala right to Inferior parietal lobule right | Peripheral | 2.89 |
| 17 | Hippocampus left | Peripheral | 17 | Putamen left to Vermis | Core | 2.9 |
| 18 | Hippocampus right | Peripheral | 18 | Rolandic operculum left to Supplementary motor area left | Peripheral | 2.91 |
| 19 | Amygdala left | Peripheral | 19 | Superior frontal gyrus (dorsal) left to Middle frontal gyrus right | Core | 2.92 |
| 20 | Amygdala right | Peripheral | 20 | Amygdala right to Angular gyrus right | Feeder | 2.92 |
| 21 | Cuneus right | Peripheral | 21 | Orbitofrontal cortex (superior) left to Middle frontal gyrus right | Feeder | 2.97 |
| 22 | Superior occipital gyrus left | Peripheral | 22 | Inferior frontal gyrus (triangular) right to Postcentral gyrus right | Peripheral | 2.98 |
| 23 | Superior occipital gyrus right | Peripheral | 23 | Postcentral gyrus right to Cerebellum left | Feeder | 2.98 |
| 24 | Middle occipital gyrus left | Core | 24 | Cuneus right to Middle occipital gyrus right | Peripheral | 2.99 |
| 25 | Middle occipital gyrus right | Peripheral | 25 | Hippocampus right to Superior occipital gyrus right | Peripheral | 3 |
| 26 | Inferior occipital gyrus left | Peripheral | 26 | Superior occipital gyrus left to Postcentral gyrus right | Peripheral | 3 |
| 27 | Postcentral gyrus left | Peripheral | 27 | Thalamus right to Vermis | Core | 3 |
| 28 | Postcentral gyrus right | Peripheral | 28 | Inferior frontal gyrus (opercular) left to Postcentral gyrus right | Peripheral | 3.02 |
| 29 | Superior parietal gyrus left | Peripheral | 29 | Hippocampus left to Angular gyrus left | Peripheral | 3.05 |
| 30 | Inferior parietal lobule left | Core | 30 | Supplementary motor area left to Postcentral gyrus right | Peripheral | 3.06 |
| 31 | Inferior parietal lobule right | Peripheral | 31 | Hippocampus right to Putamen right | Feeder | 3.06 |
| 32 | Supramarginal gyrus right | Peripheral | 32 | Hippocampus right to Supramarginal gyrus right | Peripheral | 3.07 |
| 33 | Angular gyrus left | Peripheral | 33 | Middle frontal gyrus right to Caudate right | Core | 3.07 |
| 34 | Angular gyrus right | Core | 34 | Median cingulate and paracingulate gyrus left to Postcentral gyrus right | Feeder | 3.08 |
| 35 | Caudate left | Core | 35 | Hippocampus left to Middle occipital gyrus left | Feeder | 3.11 |
| 36 | Caudate right | Core | 36 | Precentral gyrus right to Hippocampus right | Feeder | 3.12 |
| 37 | Putamen left | Core | 37 | Caudate right to Inferior temporal gyrus right | Core | 3.13 |
| 38 | Putamen right | Core | 38 | Precentral gyrus right to Cerebellum left | Core | 3.13 |
| 39 | Thalamus left | Core | 39 | Middle frontal gyrus left to Middle frontal gyrus right | Core | 3.14 |
| 40 | Thalamus right | Core | 40 | Orbitofrontal cortex (superior) left to Orbitofrontal cortex (medial) left | Peripheral | 3.14 |
| 41 | Middle temporal gyrus left | Core | 41 | Middle occipital gyrus left to Middle temporal gyrus left | Core | 3.15 |
| 42 | Inferior temporal gyrus left | Peripheral | 42 | Middle occipital gyrus left to Inferior occipital gyrus left | Feeder | 3.17 |
| 43 | Inferior temporal gyrus right | Core | 43 | Hippocampus left to Superior parietal gyrus left | Peripheral | 3.17 |
| 44 | Cerebellum left | Core | 44 | Inferior occipital gyrus left to Middle temporal gyrus left | Feeder | 3.29 |
| 45 | Cerebellum right | Core | 45 | Hippocampus right to Inferior parietal lobule right | Peripheral | 3.31 |
| 46 | Vermis | Core | 46 | Hippocampus left to Amygdala left | Peripheral | 3.34 |
|  |  |  | 47 | Hippocampus left to Cerebellum left | Feeder | 3.36 |
|  |  |  | 48 | Hippocampus right to Middle occipital gyrus right | Peripheral | 3.4 |
|  |  |  | 49 | Postcentral gyrus left to Cerebellum right | Feeder | 3.43 |
|  |  |  | 50 | Inferior frontal gyrus (triangular) left to Rolandic operculum left | Feeder | 3.44 |
|  |  |  | 51 | Inferior frontal gyrus (opercular) left to Inferior frontal gyrus (triangular) left | Feeder | 3.46 |
|  |  |  | 52 | Rolandic operculum left to Superior frontal gyrus (medial) left | Feeder | 3.49 |
|  |  |  | 53 | Thalamus right to Cerebellum left | Core | 3.51 |
|  |  |  | 54 | Putamen right to Thalamus right | Core | 3.52 |
|  |  |  | 55 | Thalamus right to Inferior temporal gyrus right | Core | 3.52 |
|  |  |  | 56 | Hippocampus right to Angular gyrus right | Feeder | 3.58 |
|  |  |  | 57 | Precentral gyrus right to Vermis | Core | 3.7 |
|  |  |  | 58 | Superior frontal gyrus (dorsal) left to Rolandic operculum left | Feeder | 3.71 |
|  |  |  | 59 | Inferior occipital gyrus left to Inferior temporal gyrus left | Peripheral | 3.72 |
|  |  |  | 60 | Hippocampus right to Postcentral gyrus right | Peripheral | 3.77 |
|  |  |  | 61 | Putamen left to Thalamus left | Core | 3.77 |
|  |  |  | 62 | Caudate right to Putamen left | Core | 3.81 |
|  |  |  | 63 | Cerebellum left to Cerebellum right | Core | 3.81 |
|  |  |  | 64 | Hippocampus left to Vermis | Feeder | 3.93 |
|  |  |  | 65 | Hippocampus left to Cerebellum right | Feeder | 3.97 |
|  |  |  | 66 | Hippocampus right to Caudate right | Feeder | 4 |

*t* = 2.9

41 nodes, 50 edges, p<0.001

| **#** | **Node** | **Node network** | **#** | **Edge** | **Edge type** | ***t*-value** |
| --- | --- | --- | --- | --- | --- | --- |
| 1 | Precentral gyrus right | Core | 1 | Putamen left to Vermis | Core | 2.9 |
| 2 | Superior frontal gyrus (dorsal) left | Core | 2 | Rolandic operculum left to Supplementary motor area left | Peripheral | 2.91 |
| 3 | Orbitofrontal cortex (superior) left | Peripheral | 3 | Superior frontal gyrus (dorsal) left to Middle frontal gyrus right | Core | 2.92 |
| 4 | Middle frontal gyrus left | Core | 4 | Amygdala right to Angular gyrus right | Feeder | 2.92 |
| 5 | Middle frontal gyrus right | Core | 5 | Orbitofrontal cortex (superior) left to Middle frontal gyrus right | Feeder | 2.97 |
| 6 | Orbitofrontal cortex (medial) left | Peripheral | 6 | Inferior frontal gyrus (triangular) right to Postcentral gyrus right | Peripheral | 2.98 |
| 7 | Inferior frontal gyrus (opercular) left | Peripheral | 7 | Postcentral gyrus right to Cerebellum left | Feeder | 2.98 |
| 8 | Inferior frontal gyrus (triangular) left | Core | 8 | Cuneus right to Middle occipital gyrus right | Peripheral | 2.99 |
| 9 | Inferior frontal gyrus (triangular) right | Peripheral | 9 | Hippocampus right to Superior occipital gyrus right | Peripheral | 3 |
| 10 | Rolandic operculum left | Peripheral | 10 | Superior occipital gyrus left to Postcentral gyrus right | Peripheral | 3 |
| 11 | Supplementary motor area left | Peripheral | 11 | Thalamus right to Vermis | Core | 3 |
| 12 | Superior frontal gyrus (medial) left | Core | 12 | Inferior frontal gyrus (opercular) left to Postcentral gyrus right | Peripheral | 3.02 |
| 13 | Median cingulate and paracingulate gyrus left | Core | 13 | Hippocampus left to Angular gyrus left | Peripheral | 3.05 |
| 14 | Hippocampus left | Peripheral | 14 | Supplementary motor area left to Postcentral gyrus right | Peripheral | 3.06 |
| 15 | Hippocampus right | Peripheral | 15 | Hippocampus right to Putamen right | Feeder | 3.06 |
| 16 | Amygdala left | Peripheral | 16 | Hippocampus right to Supramarginal gyrus right | Peripheral | 3.07 |
| 17 | Amygdala right | Peripheral | 17 | Middle frontal gyrus right to Caudate right | Core | 3.07 |
| 18 | Cuneus right | Peripheral | 18 | Median cingulate and paracingulate gyrus left to Postcentral gyrus right | Feeder | 3.08 |
| 19 | Superior occipital gyrus left | Peripheral | 19 | Hippocampus left to Middle occipital gyrus left | Feeder | 3.11 |
| 20 | Superior occipital gyrus right | Peripheral | 20 | Precentral gyrus right to Hippocampus right | Feeder | 3.12 |
| 21 | Middle occipital gyrus left | Core | 21 | Caudate right to Inferior temporal gyrus right | Core | 3.13 |
| 22 | Middle occipital gyrus right | Peripheral | 22 | Precentral gyrus right to Cerebellum left | Core | 3.13 |
| 23 | Inferior occipital gyrus left | Peripheral | 23 | Middle frontal gyrus left to Middle frontal gyrus right | Core | 3.14 |
| 24 | Postcentral gyrus left | Peripheral | 24 | Orbitofrontal cortex (superior) left to Orbitofrontal cortex (medial) left | Peripheral | 3.14 |
| 25 | Postcentral gyrus right | Peripheral | 25 | Middle occipital gyrus left to Middle temporal gyrus left | Core | 3.15 |
| 26 | Superior parietal gyrus left | Peripheral | 26 | Middle occipital gyrus left to Inferior occipital gyrus left | Feeder | 3.17 |
| 27 | Inferior parietal lobule right | Peripheral | 27 | Hippocampus left to Superior parietal gyrus left | Peripheral | 3.17 |
| 28 | Supramarginal gyrus right | Peripheral | 28 | Inferior occipital gyrus left to Middle temporal gyrus left | Feeder | 3.29 |
| 29 | Angular gyrus left | Peripheral | 29 | Hippocampus right to Inferior parietal lobule right | Peripheral | 3.31 |
| 30 | Angular gyrus right | Core | 30 | Hippocampus right to Amygdala left | Peripheral | 3.34 |
| 31 | Caudate right | Core | 31 | Hippocampus left to Cerebellum left | Feeder | 3.36 |
| 32 | Putamen left | Core | 32 | Hippocampus right to Middle occipital gyrus right | Peripheral | 3.4 |
| 33 | Putamen right | Core | 33 | Postcentral gyrus left to Cerebellum right | Feeder | 3.43 |
| 34 | Thalamus left | Core | 34 | Inferior frontal gyrus (triangular) left to Rolandic operculum left | Feeder | 3.44 |
| 35 | Thalamus right | Core | 35 | Inferior frontal gyrus (opercular) left to Inferior frontal gyrus (triangular) left | Feeder | 3.46 |
| 36 | Middle temporal gyrus left | Core | 36 | Rolandic operculum left to Superior frontal gyrus (medial) left | Feeder | 3.49 |
| 37 | Inferior temporal gyrus left | Peripheral | 37 | Thalamus right to Cerebellum left | Core | 3.51 |
| 38 | Inferior temporal gyrus right | Core | 38 | Putamen right to Thalamus right | Core | 3.52 |
| 39 | Cerebellum left | Core | 39 | Thalamus right to Inferior temporal gyrus right | Core | 3.52 |
| 40 | Cerebellum right | Core | 40 | Hippocampus right to Angular gyrus right | Feeder | 3.58 |
| 41 | Vermis | Core | 41 | Precentral gyrus right to Vermis | Core | 3.7 |
|  |  |  | 42 | Superior frontal gyrus (dorsal) left to Rolandic operculum left | Feeder | 3.71 |
|  |  |  | 43 | Inferior occipital gyrus left to Inferior temporal gyrus left | Peripheral | 3.72 |
|  |  |  | 44 | Hippocampus right to Postcentral gyrus right | Peripheral | 3.77 |
|  |  |  | 45 | Putamen left to Thalamus left | Core | 3.77 |
|  |  |  | 46 | Caudate right to Putamen left | Core | 3.81 |
|  |  |  | 47 | Cerebellum left to Cerebellum right | Core | 3.81 |
|  |  |  | 48 | Hippocampus left to Vermis | Feeder | 3.93 |
|  |  |  | 49 | Hippocampus left to Cerebellum right | Feeder | 3.97 |
|  |  |  | 50 | Hippocampus right to Caudate right | Feeder | 4 |

*t* = 3.0

35 nodes, 39 edges, p=0.001

| **#** | **Node** | **Node network** | **#** | **Edge** | **Edge type** | ***t*-value** |
| --- | --- | --- | --- | --- | --- | --- |
| 1 | Precentral gyrus right | Core | 1 | Superior occipital gyrus left to Postcentral gyrus right | Peripheral | 3 |
| 2 | Superior frontal gyrus (dorsal) left | Core | 2 | Inferior frontal gyrus (opercular) left to Postcentral gyrus right | Peripheral | 3.02 |
| 3 | Middle frontal gyrus left | Core | 3 | Hippocampus left to Angular gyrus left | Peripheral | 3.05 |
| 4 | Middle frontal gyrus right | Core | 4 | Supplementary motor area left to Postcentral gyrus right | Peripheral | 3.06 |
| 5 | Inferior frontal gyrus (opercular) left | Peripheral | 5 | Hippocampus right to Caudate right | Feeder | 3.06 |
| 6 | Inferior frontal gyrus (triangular) left | Core | 6 | Hippocampus right to Supramarginal gyrus right | Peripheral | 3.07 |
| 7 | Rolandic operculum left | Peripheral | 7 | Middle frontal gyrus right to Caudate right | Core | 3.07 |
| 8 | Supplementary motor area left | Peripheral | 8 | Median cingulate and paracingulate gyrus left to Postcentral gyrus right | Feeder | 3.08 |
| 9 | Superior frontal gyrus (medial) left | Core | 9 | Hippocampus left to Middle occipital gyrus left | Feeder | 3.11 |
| 10 | Median cingulate and paracingulate gyrus left | Core | 10 | Precentral gyrus right to Hippocampus right | Feeder | 3.12 |
| 11 | Hippocampus left | Peripheral | 11 | Caudate right to Inferior temporal gyrus right | Core | 3.13 |
| 12 | Hippocampus right | Peripheral | 12 | Precentral gyrus right to Cerebellum left | Core | 3.13 |
| 13 | Amygdala left | Peripheral | 13 | Middle frontal gyrus left to Middle frontal gyrus right | Core | 3.14 |
| 14 | Superior occipital gyrus left | Peripheral | 14 | Middle occipital gyrus left to Middle temporal gyrus left | Core | 3.15 |
| 15 | Middle occipital gyrus left | Core | 15 | Middle occipital gyrus left to Inferior occipital gyrus left | Feeder | 3.17 |
| 16 | Middle occipital gyrus right | Peripheral | 16 | Hippocampus left to Superior parietal gyrus left | Peripheral | 3.17 |
| 17 | Inferior occipital gyrus left | Peripheral | 17 | Inferior occipital gyrus left to Middle temporal gyrus left | Feeder | 3.29 |
| 18 | Postcentral gyrus left | Peripheral | 18 | Hippocampus right to Inferior parietal lobule right | Peripheral | 3.31 |
| 19 | Postcentral gyrus right | Peripheral | 19 | Hippocampus left to Amygdala left | Peripheral | 3.34 |
| 20 | Superior parietal gyrus left | Peripheral | 20 | Hippocampus left to Cerebellum left | Feeder | 3.36 |
| 21 | Inferior parietal lobule right | Peripheral | 21 | Hippocampus right to Middle occipital gyrus right | Peripheral | 3.4 |
| 22 | Supramarginal gyrus right | Peripheral | 22 | Postcentral gyrus left to Cerebellum right | Feeder | 3.43 |
| 23 | Angular gyrus left | Peripheral | 23 | Inferior frontal gyrus (triangular) left to Rolandic operculum left | Feeder | 3.44 |
| 24 | Angular gyrus right | Core | 24 | Inferior frontal gyrus (opercular) left to Inferior frontal gyrus (triangular) left | Feeder | 3.46 |
| 25 | Caudate right | Core | 25 | Rolandic operculum left to Superior frontal gyrus (medial) left | Feeder | 3.49 |
| 26 | Putamen left | Core | 26 | Thalamus right to Cerebellum left | Core | 3.51 |
| 27 | Putamen right | Core | 27 | Putamen right to Thalamus right | Core | 3.52 |
| 28 | Thalamus left | Core | 28 | Thalamus right to Inferior temporal gyrus right | Core | 3.52 |
| 29 | Thalamus right | Core | 29 | Hippocampus right to Angular gyrus right | Feeder | 3.58 |
| 30 | Middle temporal gyrus left | Core | 30 | Precentral gyrus right to Vermis | Core | 3.7 |
| 31 | Inferior temporal gyrus left | Peripheral | 31 | Superior frontal gyrus (dorsal) left to Rolandic operculum left | Feeder | 3.71 |
| 32 | Inferior temporal gyrus right | Core | 32 | Inferior occipital gyrus left to Inferior temporal gyrus left | Peripheral | 3.72 |
| 33 | Cerebellum left | Core | 33 | Hippocampus right to Postcentral gyrus right | Peripheral | 3.77 |
| 34 | Cerebellum right | Core | 34 | Putamen left to Thalamus left | Core | 3.77 |
| 35 | Vermis | Core | 35 | Caudate right to Putamen left | Core | 3.81 |
|  |  |  | 36 | Cerebellum left to Cerebellum right | Core | 3.81 |
|  |  |  | 37 | Hippocampus left to Vermis | Feeder | 3.93 |
|  |  |  | 38 | Hippocampus left to Cerebellum right | Feeder | 3.97 |
|  |  |  | 39 | Hippocampus right to Caudate right | Feeder | 4 |

*t =* 3.2

Network 1 – 10 nodes, 10 edges, p=0.009

| **#** | **Node** | **Node network** | **#** | **Edge** | **Edge type** | ***t*-value** |
| --- | --- | --- | --- | --- | --- | --- |
| 1 | Precentral gyrus right | Core | 1 | Hippocampus left to Amygdala left | Peripheral | 3.34 |
| 2 | Hippocampus left | Peripheral | 2 | Hippocampus left to Cerebellum left | Feeder | 3.36 |
| 3 | Amygdala left | Peripheral | 3 | Postcentral gyrus left to Cerebellum right | Feeder | 3.43 |
| 4 | Postcentral gyrus left | Peripheral | 4 | Thalamus right to Cerebellum left | Core | 3.51 |
| 5 | Putamen right | Core | 5 | Putamen right to Thalamus right | Core | 3.52 |
| 6 | Thalamus right | Core | 6 | Thalamus right to Inferior temporal gyrus right | Core | 3.52 |
| 7 | Inferior temporal gyrus right | Core | 7 | Precentral gyrus right to Vermis | Core | 3.7 |
| 8 | Cerebellum left | Core | 8 | Cerebellum left to Cerebellum right | Core | 3.81 |
| 9 | Cerebellum right | Core | 9 | Hippocampus left to Vermis | Feeder | 3.93 |
| 10 | Vermis | Core | 10 | Hippocampus left to Cerebellum right | Feeder | 3.97 |

Network 2 – 8 nodes, 7 edges, p=0.022

| **#** | **Node** | **Node network** | **#** | **Edge** | **Edge type** | ***t*-value** |
| --- | --- | --- | --- | --- | --- | --- |
| 1 | Hippocampus right | Peripheral | 1 | Hippocampus right to Inferior parietal lobule right | Peripheral | 3.31 |
| 2 | Middle occipital gyrus right | Peripheral | 2 | Hippocampus right to Middle occipital gyrus right | Peripheral | 3.4 |
| 3 | Postcentral gyrus right | Peripheral | 3 | Hippocampus right to Angular gyrus right | Feeder | 3.58 |
| 4 | Inferior parietal lobule right | Peripheral | 4 | Hippocampus right to Postcentral gyrus right | Peripheral | 3.77 |
| 5 | Angular gyrus right | Core | 5 | Putamen left to Thalamus left | Core | 3.77 |
| 6 | Caudate right | Core | 6 | Caudate right to Putamen left | Core | 3.81 |
| 7 | Putamen left | Core | 7 | Hippocampus right to Caudate right | Feeder | 4 |
| 8 | Thalamus left | Core |  |  |  |  |

*t =* 3.3

Network 1 –10 nodes, 10 edges, p=0.005

| **#** | **Node** | **Node network** | **#** | **Edge** | **Edge type** | ***t*-value** |
| --- | --- | --- | --- | --- | --- | --- |
| 1 | Precentral gyrus right | Core | 1 | Hippocampus left to Amygdala left | Peripheral | 3.34 |
| 2 | Hippocampus left | Peripheral | 2 | Hippocampus left to Cerebellum left | Feeder | 3.36 |
| 3 | Amygdala left | Peripheral | 3 | Postcentral gyrus left to Cerebellum right | Feeder | 3.43 |
| 4 | Postcentral gyrus left | Peripheral | 4 | Thalamus right to Cerebellum left | Core | 3.51 |
| 5 | Putamen right | Core | 5 | Putamen right to Thalamus right | Core | 3.52 |
| 6 | Thalamus right | Core | 6 | Thalamus right to Inferior temporal gyrus right | Core | 3.52 |
| 7 | Inferior temporal gyrus right | Core | 7 | Precentral gyrus right to Vermis | Core | 3.7 |
| 8 | Cerebellum left | Core | 8 | Cerebellum left to Cerebellum right | Core | 3.81 |
| 9 | Cerebellum right | Core | 9 | Hippocampus left to Vermis | Feeder | 3.93 |
| 10 | Vermis | Core | 10 | Hippocampus left to Cerebellum right | Feeder | 3.97 |

Network 2 – 5 nodes, 4 edges, p=0.046

| **#** | **Node** | **Node network** | **#** | **Edge** | **Edge type** | ***t*-value** |
| --- | --- | --- | --- | --- | --- | --- |
| 1 | Superior frontal gyrus (dorsal) left | Core | 1 | Inferior frontal gyrus (triangular) left to Rolandic operculum left | Feeder | 3.44 |
| 2 | Inferior frontal gyrus (opercular) left | Peripheral | 2 | Inferior frontal gyrus (opercular) left to Inferior frontal gyrus (triangular) left | Feeder | 3.46 |
| 3 | Inferior frontal gyrus (triangular) left | Core | 3 | Rolandic operculum left to Superior frontal gyrus (medial) left | Feeder | 3.49 |
| 4 | Rolandic operculum left | Peripheral | 4 | Superior frontal gyrus (dorsal) left to Rolandic operculum left | Feeder | 3.71 |
| 5 | Superior frontal gyrus (medial) left | Core |  |  |  |  |

Network 3 – 8 nodes, 7 edges, p=0.0012

| **#** | **Node** | **Node network** | **#** | **Edge** | **Edge type** | ***t*-value** |
| --- | --- | --- | --- | --- | --- | --- |
| 1 | Hippocampus right | Peripheral | 1 | Hippocampus right to Inferior parietal lobule right | Peripheral | 3.31 |
| 2 | Middle occipital gyrus right | Peripheral | 2 | Hippocampus right to Middle occipital gyrus right | Peripheral | 3.4 |
| 3 | Postcentral gyrus right | Peripheral | 3 | Hippocampus right to Angular gyrus right | Feeder | 3.58 |
| 4 | Inferior parietal lobule right | Peripheral | 4 | Hippocampus right to Postcentral gyrus right | Peripheral | 3.77 |
| 5 | Angular gyrus right | Core | 5 | Putamen left to Thalamus left | Core | 3.77 |
| 6 | Caudate right | Core | 6 | Caudate right to Putamen left | Core | 3.81 |
| 7 | Putamen left | Core | 7 | Hippocampus right to Caudate right | Feeder | 4 |
| 8 | Thalamus left | Core |  |  |  |  |

*t* = 3.4

Network 1 – 9 nodes, 8 edges, p=0.004

| **#** | **Node** | **Node network** | **#** | **Edge** | **Edge type** | ***t*-value** |
| --- | --- | --- | --- | --- | --- | --- |
| 1 | Precentral gyrus right | Core | 1 | Postcentral gyrus left to Cerebellum right | Feeder | 3.43 |
| 2 | Hippocampus left | Peripheral | 2 | Thalamus right to Cerebellum left | Core | 3.51 |
| 3 | Postcentral gyrus left | Peripheral | 3 | Putamen right to Thalamus right | Core | 3.52 |
| 4 | Putamen right | Core | 4 | Thalamus right to Inferior temporal gyrus right | Core | 3.52 |
| 5 | Thalamus right | Core | 5 | Precentral gyrus right to Vermis | Core | 3.7 |
| 6 | Inferior temporal gyrus right | Core | 6 | Cerebellum left to Cerebellum right | Core | 3.81 |
| 7 | Cerebellum left | Core | 7 | Hippocampus left to Vermis | Feeder | 3.93 |
| 8 | Cerebellum right | Core | 8 | Hippocampus left to Cerebellum right | Feeder | 3.97 |
| 9 | Vermis | Core |  |  |  |  |

Network 2 – 5 nodes, 4 edges, p=0.027

| **#** | **Node** | **Node network** | **#** | **Edge** | **Edge type** | ***t*-value** |
| --- | --- | --- | --- | --- | --- | --- |
| 1 | Superior frontal gyrus (dorsal) left | Core | 1 | Inferior frontal gyrus (triangular) left to Rolandic operculum left | Feeder | 3.44 |
| 2 | Inferior frontal gyrus (opercular) left | Peripheral | 2 | Inferior frontal gyrus (opercular) left to Inferior frontal gyrus (triangular) left | Feeder | 3.46 |
| 3 | Inferior frontal gyrus (triangular) left | Core | 3 | Rolandic operculum left to Superior frontal gyrus (medial) left | Feeder | 3.49 |
| 4 | Rolandic operculum left | Peripheral | 4 | Superior frontal gyrus (dorsal) left to Rolandic operculum left | Feeder | 3.71 |
| 5 | Superior frontal gyrus (medial) left | Core |  |  |  |  |

Network 3 –6 nodes, 5 edges, p=0.0014

| **#** | **Node** | **Node network** | **#** | **Edge** | **Edge type** | ***t*-value** |
| --- | --- | --- | --- | --- | --- | --- |
| 1 | Hippocampus right | Peripheral | 1 | Hippocampus right to Angular gyrus right | Feeder | 3.58 |
| 2 | Postcentral gyrus right | Peripheral | 2 | Hippocampus right to Postcentral gyrus right | Peripheral | 3.77 |
| 3 | Angular gyrus right | Core | 3 | Putamen left to Thalamus left | Core | 3.77 |
| 4 | Caudate right | Core | 4 | Caudate right to Putamen left | Core | 3.81 |
| 5 | Putamen left | Core | 5 | Hippocampus right to Caudate right | Feeder | 4 |
| 6 | Thalamus left | Core |  |  |  |  |

*t =* 3.5

Network 1 – 8 nodes, 7 edges, p=0.003

| **#** | **Node** | **Node network** | **#** | **Edge** | **Edge type** | ***t*-value** |
| --- | --- | --- | --- | --- | --- | --- |
| 1 | Precentral gyrus right | Core | 1 | Thalamus right to Cerebellum left | Core | 3.51 |
| 2 | Hippocampus left | Peripheral | 2 | Putamen right to Thalamus right | Core | 3.52 |
| 3 | Putamen right | Core | 3 | Thalamus right to Inferior temporal gyrus right | Core | 3.52 |
| 4 | Thalamus right | Core | 4 | Precentral gyrus right to Vermis | Core | 3.7 |
| 5 | Inferior temporal gyrus right | Core | 5 | Cerebellum left to Cerebellum right | Core | 3.81 |
| 6 | Cerebellum left | Core | 6 | Hippocampus left to Vermis | Feeder | 3.93 |
| 7 | Cerebellum right | Core | 7 | Hippocampus left to Cerebellum right | Feeder | 3.97 |
| 8 | Vermis | Core |  |  |  |  |

Network 2 –6 nodes, 5 edges, p=0.008

| **#** | **Node** | **Node network** | **#** | **Edge** | **Edge type** | ***t*-value** |
| --- | --- | --- | --- | --- | --- | --- |
| 1 | Hippocampus right | Peripheral | 1 | Hippocampus right to Angular gyrus right | Feeder | 3.58 |
| 2 | Postcentral gyrus right | Peripheral | 2 | Hippocampus right to Postcentral gyrus right | Peripheral | 3.77 |
| 3 | Angular gyrus right | Core | 3 | Putamen left to Thalamus left | Core | 3.77 |
| 4 | Caudate right | Core | 4 | Caudate right to Putamen left | Core | 3.81 |
| 5 | Putamen left | Core | 5 | Hippocampus right to Caudate right | Feeder | 4.00 |
| 6 | Thalamus left | Core |  |  |  |  |
