# Supplementary material for: Reduced structural connectivity in cortico-striatal-thalamic network in neonates with congenital heart disease
Source: Neuroimage Clin. 2020 Sep 15;28:102423. doi: 10.1016/j.nicl.2020.102423 (PMC7520425; doi:10.1016/j.nicl.2020.102423)
Supplement: Supplementary data 2 [file mmc2.docx]

**Supplementary results 2**

**Supplementary Table 2.** Subnetwork with reduced structural connectivity in CHD neonates after removing the outlier, its respective matched control and CHD infant. From the 29 nodes with reduced structural connectivity in infants with CHD, 18 were core nodes (out of a total of 34 core nodes, 52.9%), while 11 were peripheral nodes (out of a total of 59 peripheral nodes, 18.6%). Of the 33 edges with reduced connectivity in CHD, 12 (36.36%) were core, 15 (45.45%) were feeders and 6 (18.18%) were peripheral.

| **#** | **Node** | **Node network** | **#** | **Edge** | **Edge type** | ***t*-value** |
| --- | --- | --- | --- | --- | --- | --- |
| 1 | Superior frontal gyrus (dorsal) left | Core | 1 | Superior occipital gyrus left to Postcentral gyrus right | Peripheral | 3.11 |
| 2 | Middle frontal gyrus right | Core | 2 | Rolandic operculum left to Superior frontal gyrus (medial) left | Feeder | 3.12 |
| 3 | Orbitofrontal cortex (superior) left | Peripheral | 3 | Inferior frontal gyrus (opercular) left to Postcentral gyrus right | Peripheral | 3.12 |
| 4 | Middle frontal gyrus left | Core | 4 | Inferior parietal lobule right to Vermis | Core | 3.13 |
| 5 | Inferior frontal gyrus (opercular) left | Peripheral | 5 | Superior frontal gyrus (dorsal) left to Middle frontal gyrus right | Core | 3.14 |
| 6 | Inferior frontal gyrus (triangular) left | Core | 6 | Hippocampus left to Superior parietal gyrus left | Peripheral | 3.15 |
| 7 | Rolandic operculum left | Peripheral | 7 | Inferior frontal gyrus (triangular) left to Rolandic operculum left | Feeder | 3.16 |
| 8 | Superior frontal gyrus (medial) left | Core | 8 | Orbitofrontal cortex (superior) left to Middle frontal gyrus right | Feeder | 3.17 |
| 9 | Precentral gyrus right | Core | 9 | Hippocampus right to Thalamus right | Feeder | 3.17 |
| 10 | Hippocampus right | Peripheral | 10 | Supplementary motor area left to Postcentral gyrus right | Peripheral | 3.18 |
| 11 | Middle occipital gyrus right | Peripheral | 11 | Precentral gyrus right to Cerebellum left | Core | 3.19 |
| 12 | Postcentral gyrus right | Peripheral | 12 | Hippocampus left to Cerebellum left | Feeder | 3.21 |
| 13 | Supplementary motor area left | Peripheral | 13 | Inferior frontal gyrus (opercular) left to Inferior frontal gyrus (triangular) left | Feeder | 3.23 |
| 14 | Median cingulate and paracingulate gyrus left | Core | 14 | Caudate right to Inferior temporal gyrus right | Core | 3.25 |
| 15 | Superior occipital gyrus left | Peripheral | 15 | Precentral gyrus right to Hippocampus right | Feeder | 3.28 |
| 16 | Hippocampus left | Peripheral | 16 | Hippocampus right to Inferior parietal lobule right | Feeder | 3.33 |
| 17 | Superior parietal gyrus left | Peripheral | 17 | Hippocampus right to Middle occipital gyrus right | Peripheral | 3.34 |
| 18 | Inferior parietal lobule right | Core | 18 | Thalamus right to Cerebellum left | Core | 3.35 |
| 19 | Angular gyrus right | Core | 19 | Median cingulate and paracingulate gyrus left to Postcentral gyrus right | Feeder | 3.42 |
| 20 | Caudate right | Core | 20 | Putamen right to Thalamus right | Core | 3.43 |
| 21 | Putamen left | Core | 21 | Middle frontal gyrus left to Middle frontal gyrus right | Core | 3.44 |
| 22 | Thalamus left | Core | 22 | Hippocampus right to Angular gyrus right | Feeder | 3.46 |
| 23 | Thalamus right | Core | 23 | Superior frontal gyrus (dorsal) left to Rolandic operculum left | Feeder | 3.48 |
| 24 | Putamen right | Core | 24 | Postcentral gyrus left to Cerebellum right | Feeder | 3.51 |
| 25 | Inferior temporal gyrus right | Core | 25 | Caudate right to Putamen left | Core | 3.57 |
| 26 | Cerebellum left | Core | 26 | Cerebellum left to Cerebellum right | Core | 3.63 |
| 27 | Postcentral gyrus left | Peripheral | 27 | Thalamus right to Inferior temporal gyrus right | Core | 3.64 |
| 28 | Cerebellum right | Core | 28 | Hippocampus right to Postcentral gyrus right | Peripheral | 3.78 |
| 29 | Vermis | Core | 29 | Hippocampus left to Cerebellum right | Feeder | 3.83 |
|  |  |  | 30 | Putamen left to Thalamus left | Core | 3.85 |
|  |  |  | 31 | Hippocampus left to Vermis | Feeder | 3.90 |
|  |  |  | 32 | Precentral gyrus right to Vermis | Core | 3.99 |
|  |  |  | 33 | Hippocampus right to Caudate right | Feeder | 4.18 |
